# Syntheses and evaluation of multicaulin and miltirone-like compounds as antituberculosis agents

**DOI:** 10.1080/14756366.2017.1337758

**Published:** 2017-06-29

**Authors:** Serdar Burmaoğlu, Hatice Seçinti, Erkan Mozioğlu, Ahmet C. Gören, Ramazan Altundaş, Hasan Seçen

**Affiliations:** a Department of Chemistry, Faculty of Science, Ataturk University, Erzurum, Turkey;; b Tercan Vocational High School, Erzincan University, Erzincan, Turkey;; c Chemistry Group Laboratories, TÜBİTAK, UME, Gebze-Kocaeli, Turkey

**Keywords:** Multicaulin, miltirone, synthesis, antituberculosis

## Abstract

Four multicaulin and miltirone-like phenanthrene derivatives were synthesised and evaluated as antituberculosis agents. The crucial step of the synthesis was Pschorr coupling of 4-(3-isopropyl-4-methoxyphenyl)-2-(2-aminophenyl)ethane (**13**) to give 2-isopropyl-3-methoxy-9,10-dihydrophenanthrene (**9**) and 4-isopropyl-3-methoxy-9,10-dihydrophenanthrene (**9a**). Compound **9** was converted to multicaulin and miltirone-like phenanthrene derivatives by further reactions. The best antituberculosis activity was exhibited by 2-isopropylphenanthrene-3-ol (**11**).

## Introduction

Tuberculosis (TB) has historically been one of the most dangerous diseases. For centuries its cause was not known, but Robert Koch found in the 1880s that the real reason for TB was *Mycobacterium tuberculosis*. He also noted that 1/7 of all humans died of TB. Even today, 1/3 of all people in the world are infected with TB^1^. According to the World Health Organization, 10.4 million new TB cases were reported in 2015 and there were an estimated 1.8 million TB deaths^2^. In addition, multidrug-resistant and extremely drug-resistant TB strains are still considered as a problem for medicine to overcome^3^. The mortality rate for TB remains high despite the availability of antibiotics (isoniazide, ethambutol, pyrazinamide, rifampicin, and streptomycin) for its treatment^4^. Recently, Kimpe et al. synthesised some new heterocycles and reported their strong antimycobacterial activities^5–8^. In this context, there is an urgent need to develop new drugs to overcome the newer forms of TB. Conventionally, natural products have played an important role in the development of new drugs for the treatment of many diseases^9^. Today, nearly one-third of the top-selling drugs in the world are natural products or their synthetic derivatives^10^. Plants of the genus *Salvia*, which is a member of the family Lamiaceae, are used worldwide as conventional medicines with various biological activities such as antibacterial, antioxidant, antidiabetic, antitumor, and antituberculous^11^^,^^12^. Ulubelen et al. reported that these species have also been used for the treatment of many ailments, including haemorrhaging, menstrual disorders, miscarriage, heart disease, and hepatitis^13^.

**Figure 1.**
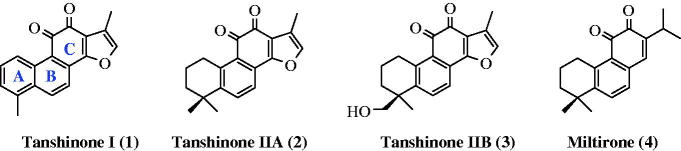
Structures of abietane-type diterpenes **1–4**, which include a quinone moiety in the C-ring.

Phytochemical studies of *Salvia* species have been conducted by many research groups, and compounds including diterpenoids, sesquiterpenoids, sesterterpenoids, steroids, and polyphenols have been identified as key constituents of these plants. The diterpenoids found in these species, particularly tanshinones (**1**, **2**, **3**) and miltirone (**4**), which are 20-norditerpenes with an abietane-type skeleton that includes a quinone moiety in the C-ring (Figure 1), have been widely studied^14^^,^^15^. These compounds behave as antioxidants against lipid peroxidation *in vitro* and *in vivo*. A study showed the potential anticancer activity of tanshinone IIA *in vitro* and *in vivo* against both estrogen receptor (ER)-positive and ER-negative breast cancers^16^. Chang et al. also reported the most potential activity of miltirone among 10 diterpene quinones isolated from *Salvia miltiorrhiza* in the central benzodiazepine receptor binding assay (IC_50_ = 0.3 pM)^17^.

**Figure 2.**
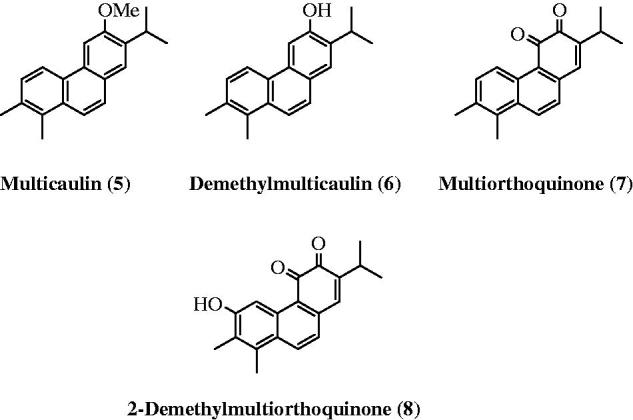
Structures of aromatic norabietanes **5–8**.

Ulubelen et al.^13^ isolated an additional four aromatic norabietanes (**5**–**8**) with structural and biological properties similar to those of the highly active diterpenoids tanshinones and miltirone (Figure 2). These compounds exhibit strong antituberculous activity, with minimal inhibitory concentration (MIC) values ranging from 0.46 to 7.3 μg/ml^13^. Previously, we achieved the first total synthesis of the antituberculous agents multicaulin (**5**) and *O*-demethylmulticaulin (**6**) based on an oxidative photochemical reaction of the corresponding stilbene^18^.

**Figure 3.**
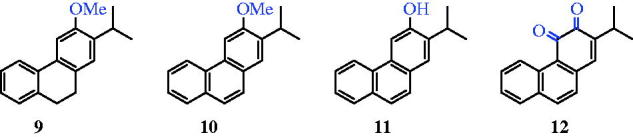
Four C-ring functionalised abietane-like compounds (**9–12**).

Herein, we describe a new method for four C-ring functionalised abietane-like compounds (**9–12**) based on a direct chemical synthesis and the evaluation of their antimycobacterial activities (Figure 3). To the best of our knowledge, among compounds **9–12**, there was only one article describing a synthesis of phenanthrene derivative **10** in 1957, in which Sengupta et al.^19^ prepared compound **10** starting from ethyl 2-methoxybenzoate in 11 steps and with a yield of less than 1%.

**Scheme 1.**
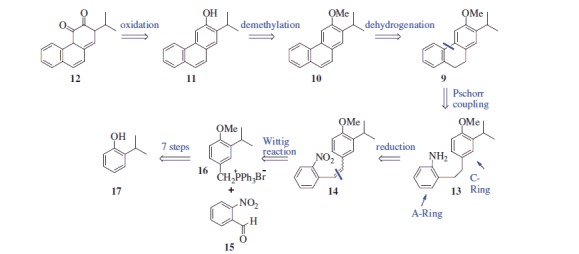
Retrosynthetic analysis for compounds **9**–**12**.

A retrosynthetic analysis of abietane-like compounds **9–12** is shown in Scheme 1. The key step in this strategy is chosen as the connection of the A and C rings via the Pschorr reaction of **13**.

## Experimental

### General experimental procedures

Commercially available reagents and solvents were of analytical grade or were purified by standard procedures prior to use. Reactions were monitored via thin-layer chromatography (TLC). The ^1^H NMR and ^13^C NMR spectra were recorded on a 200(50) MHz and 400(100) MHz Varian spectrometer using CDCl_3_. Chemical shifts (δ) are reported in parts per million (ppm) relative to either a tetramethylsilane (TMS) internal standard or solvent signals. Interchangeable hydrogens and carbons were assigned with the letter. High-resolution mass spectrometry (HRMS) were recorded in Bruker Daltonics microTOF-Q instrument by using atmospheric pressure chemical ionization-electrospray ionization (APCI-ESI) ion source. Column chromatography was performed on silica gel 60 (70–230 mesh ASTM), and TLC was carried out on silica gel (254–366 mesh ASTM). The purity of biologically tested compounds was determined by Q NMR (purity >98%)^20–23^.

#### 4-Bromo-2-isopropylanisole (19)

Compound **19** was synthesised according to the procedure reported in our previous study^18^.

#### 3-Isopropyl-4-methoxybenzonitrile (20)

CuCN (7.60 g; 84.8 mmol) was placed into a 250-ml flask and a solution of 4-bromo-2-isopropylanisole (**19**) (6.48 g, 28.3 mmol) in dimethylformamide (DMF) (40 ml) was added under N_2_. The reaction mixture was stirred at 140 °C for 12 h. The reaction mixture was allowed to cool to rt and EtOAc (100 ml) was added. The mixture was washed with a 10% solution of FeCl_3_ (150 ml). The organic layer was dried with MgSO_4_. The solvent was removed under reduced pressure to afford 3-isopropyl-4-methoxybenzonitrile (**20**) as a brown liquid (4.44 g, 90%). *R_f_* = 0.5 (1:9 EtOAc-hexanes).

^1^H NMR (200 MHz, CDCl_3_) δ 7.46 (dd, 1H, H-6, *J* = 8.6 Hz, *J* = 2.1 Hz); 7.45 (d, 1H, H-2, *J* = 2.1 Hz); 6.87 (d, 1H, H-5, *J* = 8.6 Hz); 3.87 (s, 3H, OMe); 3.29 (septet, 1H, CHMe_2_, *J* = 6.8 Hz); 1.18 (d, 6H, CHMe_2_, *J* = 6.8 Hz).

^13^C NMR (50 MHz, CDCl_3_) δ 159.1 (C-4); 138.3 (C-3); 131.4 (C-2 or C-6); 129.9 (C-2 or C-6); 119.6 (CN); 110.5 (C-5); 103.7 (C-1); 55.5 (OMe); 26.6 (CHMe_2_); 22.1 (CHMe_2_).

#### Ethyl 3-isopropyl-4-methoxybenzoate (21)

To a solution of **20** (4.22 g, 24.1 mmol) in EtOH (50 ml), H_2_SO_4_ (98%, 7.16 ml, 145 mmol) was carefully added. The reaction mixture was refluxed for 12 h. The mixture was then allowed to cool to rt, and the excess EtOH was removed under reduced pressure. The crude product was dissolved in EtOAc (100 ml) and washed with H_2_O (2 × 100 ml). The organic layer was dried with MgSO_4_, and the solvent was removed under reduced pressure to afford ethyl 3-isopropyl-4-methoxybenzoate (**21**) as a brown liquid (4.50 g, 84%). Compound **21** was used in the next step without further purification.

^1^H NMR (200 MHz, CDCl_3_) δ 7.90 (s, 1H, H-2); 7.88 (d, 1H, H-6, *J* = 8.3 Hz); 6.82 (d, 1H, H-5, *J* = 8.3 Hz); 4.34 (q, 2H, OCH_2_CH_3_, *J* = 7.0 Hz); 3.85 (s, 3H, OMe); 3.31 (septet, 1H, CHMe_2_, *J* = 7.0 Hz); 1.37 (t, 3H, OCH_2_CH_3_, *J* = 7.0 Hz); 1.22 (d, 6H, CHMe_2_, *J* = 7.0 Hz).

^13^C NMR (50 MHz, CDCl_3_) δ 166.5 (CO); 160.5 (C-4); 136.8 (C-1); 128.8 (C-6); 127.6 (C-2); 122.6 (C-3); 109.5 (C-5); 60.3 (OCH_2_CH_3_); 55.3 (OMe); 26.7 (CHMe_2_); 22.2 (CHMe_2_); 14.2 (OCH_2_CH_3_).

#### 3-Isopropyl-4-methoxybenzyl alcohol (22)^24^

To a suspension of LiAlH_4_ (2.37 g, 62.4 mmol) in tetrahydrofuran (THF) (20 ml), a solution of **21** (4.11 g, 18.5 mmol) in THF (20 ml) was added dropwise at 0 °C under N_2_. The reaction mixture was stirred for 4 h at rt. After monitoring with TLC, the reaction mixture was cooled to 0 °C, and a saturated solution of NH_4_Cl (10 ml) was added, followed by the addition of EtOAc (30 ml). The precipitate was filtered, and then the organic phase was washed with water (2 × 20 ml) and dried with MgSO_4_. Finally, the solvent was removed under reduced pressure to yield 3-isopropyl-4-methoxybenzyl alcohol (**22**) as a yellow liquid (3.03 g, 91%). *R_f_* = 0.23 (1:4 EtOAc-hexanes).

^1^H NMR (200 MHz, CDCl_3_) δ 7.24 (d, 1H, H-2, *J_2,6_* = 2.0 Hz); 7.18 (dd, 1H, H-6, *J* = 8.1 Hz, *J* = 2.0 Hz); 6.84 (d, 1H, H-5, *J* = 8.1 Hz); 4.59 (s, 2H, CH_2_OH); 3.85 (s, 3H, OMe); 3.36 (septet, 1H, CHMe_2_, *J* = 7.0 Hz); 2.43 (bs, 1H, OH); 1.26 (d, 6H, CHMe_2_, *J* = 7.0 Hz).

^13^C NMR (50 MHz, CDCl_3_) δ 156.2 (C-4); 137.0 (C-3); 132.8 (C-1); 125.4 (C-2 or C-6); 125.2 (C-2 or C-6); 110.2 (C-5); 65.0 (CH_2_OH); 55.3 (OMe); 26.6 (CHMe_2_); 22.5 (CHMe_2_).

The ^1^H NMR and ^13^C NMR spectra are in agreement with the reported data of Burnell and Caron^24^.

#### 3-Isopropyl-4-methoxybenzyl bromide (23)^24^

To a solution of **22** (3.50 g, 19.4 mmol) in CH_2_Cl_2_ (50 ml), PBr_3_ (5.79 g, 2.01 ml, 21.4 mmol) was added dropwise at 0 °C. The reaction mixture was stirred for 12 h at rt. After monitoring with TLC, water (50 ml) was added to the mixture. The organic layer was then separated and dried with MgSO_4_, and the solvent was removed under reduced pressure to afford 3-isopropyl-4-methoxybenzylbromide (**23**) as a red liquid (4.35 g, 92%).

^1^H NMR (200 MHz, CDCl_3_) δ 7.29 (d, 1H, H-2, *J* = 2.2 Hz); 7.26 (dd, 1H, H-6, *J* = 8.2 Hz, *J* = 2.2 Hz); 6.84 (d, 1H, H-5, *J* = 8.2 Hz); 4.56 (s, 2H, CH_2_Br); 3.86 (s, 3H, OMe); 3.36 (septet, 1H, CHMe_2_, *J* = 6.9 Hz); 1.28 (d, 6H, CHMe_2_, *J* = 6.9 Hz).

^13^C NMR (50 MHz, CDCl_3_) δ 156.9 (C-4); 137.4 (C-3); 129.7 (C-1); 127.4 (C-2 or C-6); 127.1 (C-2 or C-6); 110.4 (C-5); 55.4 (OMe); 34.5 (CH_2_Br); 26.8 (CHMe_2_); 22.5 (CHMe_2_).

The ^1^H NMR and ^13^C NMR spectra are in agreement with the reported data of Burnell and Caron^24^.

#### (3-isopropyl-4-methoxybenzyl)triphenylphosphonium bromide (16)

To a solution of **23** (3.41 g, 14.0 mmol) in CH_3_CN (100 ml), PPh_3_ (4.04 g, 15.4 mmol) was added, and the reaction mixture was refluxed for 24 h. After monitoring by TLC, the solvent was removed under reduced pressure to afford (3-isopropyl-4-methoxy)triphenylphosphonium bromide (**16**) as a white solid (7.06 g). The salt was used for the next step without further purification.

#### (E/Z)-1-(3-isopropyl-4-methoxyphenyl)-2-(2-nitrophenyl)ethene (14)

To a suspension of NaH (2.39 g, 99.6 mmol) in CH_2_Cl_2_ (20 ml) under N_2_, **16** (7.06 g, 14.0 mmol) in CH_2_Cl_2_ (40 ml) was added at 0 °C, and the mixture was stirred for 15 min at the same temperature. After the mixture was stirred for 15 min, 2-nitrobenzaldehyde (**15**) (2.51 g, 16.6 mmol) in CH_2_Cl_2_ (40 ml) was added to the mixture, and the reaction mixture was stirred for 16 h at rt. After monitoring with TLC, a saturated solution of NH_4_Cl (20 ml) was added dropwise to the mixture to quench the excess NaH. The organic layer was separated and dried with MgSO_4_. The solvent was removed under reduced pressure, and the crude product was purified by silica gel chromatography (1:4 EtOAc-hexanes) to yield an isomeric mixture of stilbene (*E/Z*)-**14** (4.0 g) as a yellow liquid. This mixture was used for the next step without further purification.

^1^H NMR of (*E*)-isomer (400 MHz, CDCl_3_) δ 7.94 (dd, 1H, H-3''^a^, *J* = 8.1 Hz, *J* = 1.5 Hz); 7.76 (dd, 1H, H-6''^a^, *J* = 8.1 Hz, *J* = 1.5 Hz); 7.57 (bt, 1H, H-5''^b^, *J* = 8.1 Hz); 7.46 (d, 1H, H-2, *J* = 16.1 Hz); 7.38 (s, 1H, H-2'); 7.37 (d, 1H, H-6', *J* = 9.1 Hz); 7.36 (bt, 1H, H-4''^b^, *J* = 8.1 Hz); 7.08 (d, 1H, H-1, *J* = 16.1 Hz); 6.86 (d, 1H, H-5', *J* = 9.1 Hz); 3.86 (s, 3H, OMe); 3.33 (septet, 1H, CHMe_2_, *J* = 6.6 Hz); 1.24 (d, 6H, CHMe_2_, *J* = 6.6 Hz).

HRMS *m/z* (by using APCI-ESI ion source) 298. 1436 [M + H] + (calcd. for C_18_H_19_NO_3_ 298.1438).

#### 1-(3-Isopropyl-4-methoxyphenyl)-2-(2-aminophenyl)ethane (13)

Pd-C (400 mg) was placed into a 250-ml flask and cooled MeOH (40 ml) was carefully added. A solution of **14** (4.00 g) in MeOH (60 ml) was then added to the mixture. The reaction was stirred under H_2_ (balloon, 1 atm) for 2 h at rt. The catalyst was filtered and the solvent was removed under reduced pressure to afford 1-(3-isopropyl-4-methoxyphenyl)-2-(2-aminophenyl)ethane (**13**) (3.23 g, 12.0 mmol) as a pale brown liquid. Overall yield from **23** to **13** was 78%.

^1^H NMR (400 MHz, CDCl_3_) δ 7.16–7.01 (m, 4H, H-4'', H-6'', H-2', H-6'); 6.86 (d, 1H, H-5', *J* = 9.0 Hz); 6.84 (dt, 1H, H-5'', *J* = 7.4 Hz, *J* = 1.2 Hz); 6.74 (bd, 1H, H-3'', *J* = 7.8 Hz); 3.89 (s, 3H, OMe); 3.44 (bs, 2H, NH_2_); 3.40 (septet, 1H, CHMe_2_, *J* = 7.0 Hz); 3.00–2.83 (m, 4H, A_2_B_2_ system, 2xH-1, 2xH-2); 1.29 (d, 6H, CHMe_2_, *J* = 7.0 Hz).

^13^C NMR (100 MHz, CDCl_3_,) δ 155.1 (C-4'); 144.2 (C-2''); 136.8 (s); 133.6 (s); 129.4 (d); 127.0 (d); 126.3 (s); 126.2 (d); 126.1 (d); 118.8 (d); 115.6 (d); 110.4 (d); 55.4 (OMe); 34.7 (C-1^a^); 33.6 (C-2^a^); 26.6 (CHMe_2_); 22.6 (CHMe_2_).

HRMS *m/z* (by using APCI-ESI ion source) 270.1850 [M + H] + (calcd. for C_18_H_23_NO 270.1852).

#### 2-Isopropyl-3-methoxy-9,10-dihydrophenanthrene (9)

To a solution of **13** (0.46 g, 1.71 mmol) in acetone (20 ml), H_2_SO_4_ (98%, 0.18 ml, 3.5 mmol) and isopentyl nitrite (0.40 g, 0.45 ml, 3.41 mmol) were added carefully at 0 °C. The reaction mixture was stirred at 0–10 °C for 1 h; then H_2_O (10 ml) was added and the mixture was stirred further for 1 h. After 2 h, a saturated NaHSO_3_ solution (5 ml) was added to the mixture, and the mixture was poured into H_2_O (50 ml). The mixture was extracted with CHCl_3_ (2 × 50 ml), and the combined organic layers were separated and dried with MgSO_4_. The solvent was removed under reduced pressure, and the crude product was purified by silica gel chromatography (3:7 EtOAc-hexanes) to yield 2-isopropyl-3-methoxy-9,10-dihydrophenanthrene (**9**) (77 mg, 18%) and 4-isopropyl-3-methoxy-9,10-dihydrophenanthrene (**9a**) (25 mg, 6%).

^1^H NMR of 2-isopropyl-3-methoxy-9,10-dihydrophenanthrene (**9**) (400 MHz, CDCl_3_) δ 7.71 (bd, 1H, H-5, *J* = 7.7 Hz); 7.29 (ddd, quasi dt, 1H, H-6, *J* = 7.7 Hz, *J* = 6.9 Hz, *J* = 1.8 Hz); 7.23 (s, 1H, H-1); 7.22–7.18 (m, 2H, H-7 and H-8); 7.01 (s, 1H, H-4); 3.91 (s, 3H, OMe); 3.33 (septet, 1H, CHMe_2_, *J* = 7.0 Hz); 2.88–2.78 (m, A_2_B_2_ system, 4H, 2xH-9 and 2xH-10); 1.24 (d, 6H, CHMe_2_, *J* = 7.0 Hz).

^13^C NMR of 2-isopropyl-3-methoxy-9,10-dihydrophenanthrene (**9**) (100 MHz, CDCl_3_) δ 156.0 (C-5); 137.3 (s); 136.5 (s); 134.7 (s); 132.5 (s); 129.5 (s); 128.1 (d); 127.0 (d); 126.8 (d); 125.9 (d); 123.3 (d); 106.0 (d); 55.7 (OMe); 29.5 (C-9^a^); 28.3 (C-10^a^); 26.7 (CHMe_2_); 22.8 (CHMe_2_).

^1^H NMR of 4-isopropyl-3-methoxy-9,10-dihydrophenanthrene (**9a**) (400 MHz, CDCl_3_) δ 7.49 (bd, 1H, H-5, *J* = 7.7 Hz); 7.29–7.19 (m, 3H, H-6, H-7, H-8); 7.06 (d, 1H, H-1, *J* = 8.2 Hz); 6.77 (d, 1H, H-2, *J* = 8.2 Hz); 3.85 (s, 3H, OMe); 3.70 (septet, 1H, CHMe_2_, *J* = 7.0 Hz); 2.73–2.63 (m, A_2_B_2_ system, 4H, 2xH-9 and 2xH-10); 1.41 (d, 6H, CHMe_2_, *J* = 7.0 Hz).

#### 2-isopropyl-3-methoxyphenanthrene (10)

To a solution of **9** (50 mg, 0.20 mmol) in toluene (2 ml), a solution of 2,3-dichloro-5,6-dicyano-1,4-benzoquinone (DDQ) (49.5 mg, 0.22 mmol) in toluene (5 ml) was added. The reaction mixture was refluxed for 24 h. After monitoring by TLC, the mixture was allowed to reach rt and then the solvent was removed under reduced pressure. The crude product was purified by TLC eluting with hexane to yield 2-isopropyl-3-methoxyphenanthrene (**10**) (21 mg, 42%) as a colourless liquid and 3-methoxy-2-(prop-1-en-2-yl)phenanthrene (**10a**) (14 mg, 28%) as a colourless liquid.

^1^H NMR of 2-isopropyl-3-methoxyphenanthrene (**10**) (400 MHz, CDCl_3_) δ 8.60 (bd, 1H, H-5, *J* = 8.1 Hz); 7.98 (s, 1H, H-1); 7.87 (d, 1H, H-8, *J* = 7.7 Hz); 7.69 (s, 1H, H-4); 7.68 (d, 1H, H-9^a^, *J* = 8.8 Hz); 7.62 (bt, 1H, H-6 overlapped with H-10); 7.61 (d, 1H, H-10^a^, *J* = 8.8 Hz); 7.56 (dt, 1H, H-7, *J* = 7.7 Hz, *J* = 1.1 Hz); 4.08 (s, 3H, OMe); 3.48 (septet, 1H, CHMe_2_, *J* = 6.6 Hz); 1.35 (d, 6H, CHMe_2_, *J* = 6.6 Hz).

^13^C NMR of 2-isopropyl-3-methoxyphenanthrene (**10**) (100 MHz, CDCl_3_) δ 156.6 (C-3); 138.3 (s); 132.1 (s); 129.8 (s); 129.5 (s); 128.6 (d); 126.7 (s); 126.6 (d); 126.1 (d); 125.9 (d); 125.7 (d); 124.4 (d); 122.4 (d); 101.8 (d); 55.5 (OMe); 27.1 (isopropyl CH); 22.8 (2xCH_3_).

^1^H NMR of 3-methoxy-2-(prop-1-en-2-yl)phenanthrene (**10a**) (400 MHz, CDCl_3_) δ 8.60 (d, 1H, H-5, *J* = 8.4 Hz); 8.00 (s, 1H, H-1); 7.87 (d, 1H, H-8, *J* = 7.7 Hz); 7.70 (s, 1H, H-4); 7.67 (d, 1H, H-9, *J* = 9.2 Hz); 7.63 (t, 1H, H-6 overlapped with H-10); 7.62 (d, 1H, H-10, *J* = 9.2 Hz); 7.58 (t, 1H, H-7, *J* = 7.7 Hz); 5.26 (bs, 1H, C = CH_2_); 5.22 (bs, 1H, C = CH_2_); 4.07 (s, 3H, OMe); 2.22 (bs, 3H, =C-CH_3_).

#### 2-Isopropylphenanthren-3-ol (11)

To a solution of **10** (100 mg, 0.40 mmol) in CH_2_Cl_2_ (30 ml), BBr_3_ (110 mg, 0.04 ml, 0.44 mmol) was added dropwise under N_2_ at 0 °C. The reaction mixture was stirred at rt for 12 h; then MeOH (10 ml) was added to the mixture and the solvent was removed under reduced pressure. The crude product was dissolved in EtOAc (30 ml) and washed with H_2_O (2 × 30 ml). The organic layer was dried with Na_2_SO_4_ and removed under reduced pressure to afford 2-isopropylphenanthren-3-ol (**11**) (88 mg, 93%) as a yellow solid. *R_f_* = 0.70 (1:4 EtOAc-hexanes), Mp =141–143 °C.

^1^H NMR (400 MHz, CDCl_3_) δ 8.48 (d, 1H, H-5, *J* = 7.6 Hz); 7.94 (s, 1H, H-1); 7.85 (bd, 1H, H-8, *J* = 8.0 Hz); 7.70 (s, 1H, H-4); 7.67 (A part of AB system, d, 1H, H-9, *J* = 9.3 Hz); 7.59 (B part of AB system, d, 1H, H-10, *J* = 9.3 Hz); 7.59–7.52 (m, 2H, H-6 and H-7); 5.19 (s, 1H, OH); 3.40 (septet, 1H, CH(CH_3_)_2_, *J* = 7.0 Hz); 1.40 (d, 3H, CH_3_, *J* = 7.0 Hz); 1.39 (d, 3H, CH_3_, *J* = 7.0 Hz).

^13^C NMR (100 MHz, CDCl_3_) δ 152.5 (C-3); 136.0 (s); 132.0 (s); 129.7 (s); 129.4 (s); 128.5 (d); 127.0 (s); 126.6 (d); 126.2 (d); 126.2 (d); 126.0 (d); 124.4 (d); 122.5 (d); 106.9 (d); 27.5 (CHMe_2_); 22.6 (2xCHMe_2_).

HRMS *m/z* (by using APCI-ESI ion source) 237.1272 [M + H] + (calcd. for C_17_H_16_O 237.1274).

#### 2-Isopropylphenanthren-3,4-dione (12)

To a solution of **11** (100 mg, 0.42 mmol) in CH_2_Cl_2_ (20 ml), Dess–Martin periodinane (269 mg, 0.63 mmol) was added dropwise at 0 °C. The reaction mixture was stirred at rt for 12 h. After monitoring by TLC, the reaction mixture was washed with 1 M NaOH solution (20 ml). The organic layer was separated and dried with Na_2_SO_4_, and the solvent was removed under reduced pressure to afford 2-isopropylphenanthren-3,4-dione (**12**) (64.1 mg, 61%) as a red liquid. *R_f_* = 0.40 (1:4 EtOAc-hexanes).

^1^H NMR (400 MHz, CDCl_3_) δ 9.37 (d, 1H, H-5, *J* = 8.6 Hz); 8.08 (d, 1H, H-8, *J* = 8.6 Hz); 7.80 (bd, 1H, H-10, *J* = 8.2 Hz); 7.69 (ddd, quasi bt, 1H, H-6 ^b^, *J* = 8.6 Hz, *J* = 6.9 Hz, *J* = 1.4 Hz); 7.52 (ddd, quasi bt, 1H, H-7 ^b^, *J* = 8.6 Hz, *J* = 6.9 Hz, *J* = 0.9 Hz); 7.35 (d,1H, H-9, *J* = 8.2 Hz); 7.18 (d, 1H, H-1, *J* = 0.9 Hz); 3.07 (septet, 1H, CH(CH_3_)_2_, *J* = 6.9 Hz); 1.21 (d, 6H, CH(CH_3_)_2_, *J* = 6.9 Hz).

^13^C NMR (100 MHz, CDCl_3_) δ 182.3 (s); 181.5 (s); 146.9 (s); 139.7 (d); 137.5 (d); 134.3 (s); 132.5 (s); 131.3 (d); 129.1 (d); 127.7 (d); 127.1 (d); 126.9 (d); 125.0 (s); 27.4 (d); 21.7 (q).

HRMS *m/z* (by using APCI-ESI ion source) 251.1066 [M + H] + (calcd. for C_17_H_14_O_2_ 251.1067).

## Results and discussion

### Synthesis

**Scheme 2.**
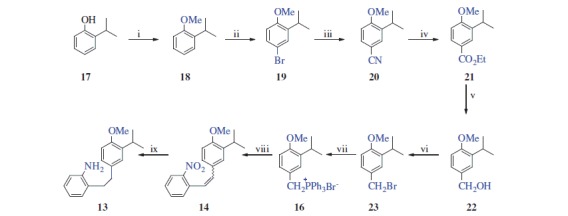
Reaction conditions and reagents: (i) Me_2_SO_4_, NaOH (aq), 90 °C, 4 h, 93%; (ii) LiBr/(NH_4_)_2_Ce(NO_3_)_6_, CH_3_CN, 20 °C, 2 h, 97%; (iii) CuCN, DMF, 140 °C, 12 h, 90%; (iv) EtOH, H_2_SO_4_, reflux, 12 h, 84%; (v) LiAlH_4_, THF, 0 °C, 4 h, 91%; (vi) PBr_3_, DCM, 0 °C, 12 h, 92%; (vii) PPh_3_, CH_3_CN, reflux, 24 h; (viii) NaH, DCM, 0 °C, 15 min; and 2-nitrobenzaldehyde (**15**), 20 °C, rt, 16 h; and (ix) H_2_, Pd/C, MeOH, 20 °C, 2 h.

2-Isopropylphenol (**17**) was used as the starting material for the preparation of **13**. The synthesis of **19** with a sequence of **17** → **18** → **19** was described in our previous study in two steps^18^. Following our previous procedure, compound **17** was converted to **19** by *O*-methylation followed by selective bromination with CAN/LiBr. Preparation of benzyl bromide **23** from compound **19** was as described by Burnell and Caron^24^. Following this procedure with a slight modification, we prepared compound **23**. For this purpose, treatment of compound **19** with CuCN provided benzonitrile **20** in 90% yield, which was then esterified with EtOH to give ester **21** in the presence of H_2_SO_4_ (94% yield). Reduction of ester **21** using LiAlH_4_ gave alcohol **22** (81% yield). Treatment of this alcohol with PBr_3_ afforded benzyl bromide **23** in 92% yield. Phosphonium salt **16** was prepared from benzyl bromide **23** by reaction with PPh_3_ and used in the next step without further purification. The Wittig reaction of **16** with 2-nitrobenzaldehyde (**15**) resulted in an (*E/Z*) mixture of stilbene **14**, which was then hydrogenated on Pd-C to give aniline **13** in 86% overall yield in three steps from **23** (Scheme 2).

**Scheme 3.**
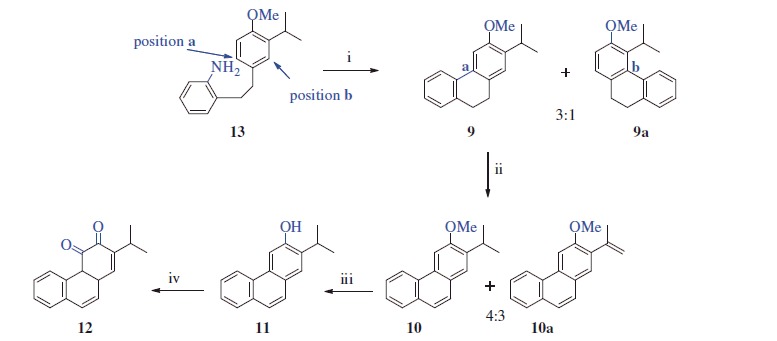
Synthesis of **11** and **12**^a^. ^a^Reaction conditions and reagents: (i) isopentyl nitrite, H_2_SO_4_, acetone, 0–10 °C, 2 h, 24%; (ii) DDQ, toluene, reflux, 24 h, 70%; (iii) BBr_3_, DCM, N_2_ atm., 0 °C, 12 h, 93%; (iv) DMP, DCM, 0 °C, 12 h, 61%.

The most important step in our synthetic strategy was the conversion of amine **13** to tricyclic compound **9** via an intramolecular Pschorr coupling reaction. The Pschorr reaction has been known for over a century and proceeds through aryldiazonium salt of biaryl to give tricyclic arenes^25^. In this context, Caronna et al. synthesised 4-methoxy-9,10-dihydrophenanthrene via Pschorr coupling^26^. By a similar approach, the intramolecular Pschorr reaction of **13** gave the desirable compound **9** along with side product **9a** in a yield of 24% (**9:9a** = 3:1). Thus, after diazotisation of **13**, two different ring closure reactions occurred at position a and position b (Scheme 3). In this work, we suppose that amine **13** should give a phenanthrene ring by a similar approach. Assignment of the structures of the two isomers was performed via ^1^H NMR analysis. The ^1^H NMR spectrum of **9** displayed two singlets for the H-1 (δ 7.23 ppm) and H-4 (δ 7.01 ppm) protons. On the other hand, the ^1^H NMR spectrum of **9a** displayed an AB system for H-1 and H-2 at δ 7.06 and δ 6.77 ppm, respectively. To obtain phenanthrene **10**, compound **9** was treated with powerful oxidant DDQ. As a result of oxidation of **9**, the desired product **10** was obtained in a yield of 42% along with a 29% yield of side product **10a** (4:3 ratio, Scheme 3). This mixture was successfully separated using column chromatography, and their structures were evaluated by NMR spectra. Signals for the CH_2_CH_2_ at the C-9 and C-10 positions that were present in the ^1^H NMR spectrum of **9** were not detected in the ^1^H NMR spectra of **10** and **10a**, indicating that both compounds were aromatised. In addition, the ^1^H NMR spectrum of **10a** displayed two broad singlets for olefinic protons at δ 5.26 and δ 5.22 ppm, indicating that the isopropyl group was also oxidised during the reaction. The formation of **10a** is thought to proceed via a stable carbenium ion at C-2 of isopropyl (Scheme 3). Next, *O*-demethylation of **10** via treatment with BBr_3_ yielded phenanthrene **11** in 93% yield. In the ^1^H NMR spectrum of **11**, no singlet for the methoxy protons (δ 4.07 in the ^1^H NMR spectrum of **10**) was detected. Finally, compound **12** was prepared in 61% yield via the Dess–Martin periodinane oxidation of **11**. The ^1^H NMR spectrum of **12** displayed four doublets for H-5 (δ 9.37), H-8 (δ 8.08), H-9 (δ 7.35) and H-10 (δ 7.80); two quasi-broad triplets for H-6 (δ 7.69) and H-7 (δ 7.52); and one doublet for H-1 (δ 7.18) with ^4 ^*J* = 0.9 Hz (Scheme 3).

### Biological assessment – antimycobacterial activity

The chemical compounds were tested against standard bacterial strains *Mycobacterium smegmatis* ATCC14468, *Mycobacterium bovis*, *Mycobacterium szulgai* ATCC35799, *Mycobacterium gastri* ATCC15754, *Mycobacterium simiae* ATCC25275 and *M. tuberculosis* H37Ra for the determination of their antimycobacterial activities (Table 1). The MIC studies were performed as described by Sokmen et al.^27^ In brief, 100 μl of Middlebrook 7H9 Broth was dispersed into the wells, and 100 μl of the chemical compounds was added into the first wells. After being pipetted, 100 μl of the first wells was transferred to the second wells. In this way, serial dilutions were performed. After dilutions, 95 μl of Middlebrook 7H9 Broth was added into the wells, and then 5 μl of the inoculum (with 0.5 McFarland turbidity) was added. Thus, the final volume of each well was 200 μl. The concentration range of chemical compounds was 0–1000 μl/ml. Streptomycin was used as a positive control. Since the chemical compounds were dissolved in ethanol, it was used as a negative control. The plates were incubated at 37 °C until bacterial growth was observed in the wells including 0 μl/ml chemical compounds. The lowest concentrations of the chemical compounds that inhibited the growth of the bacteria were considered as the MIC values. Each experiment was replicated three times for the determination of the MIC.

**Table 1.** *In vitro* antimycobacterial activity of synthesised compounds (**9**–**12**) expressed as the minimum inhibitory concentration (μm).

Chemical compounds	9	10	11	12	Streptomycin
*M. smegmatis* ATCC14468	>991.50	>999.40	>1058.80	499.98	2.68
*M. bovis* ATCC35734	991.50	249.90	>1058.80	>999.96	0.67
*M. szulgai* ATCC35799	>991.50	62.49	66.20	62.52	1.30
*M. gastri* ATCC15754	>991.50	62.49	33.03	62.52	0.17
*M. simiae* ATCC25275	>991.50	249.90	66.20	249.99	2.68
*M. tuberculosis* (H37Ra) ATCC25177	>991.50	NA	8.26	>999.96	0.84

NA: No assay. Streptomycin: Positive control.

## Conclusions

In this study, stilbene **14** was prepared from 2-isopropylphenol in eight steps in a total yield of 49%. Catalytic hydrogenation of **14** gave **13**, which underwent a Pschorr coupling reaction via diazotisation to afford dihydrophenanthrenes **9** and **9a**. Oxidation of **9** with DDQ gave **10** as the main product. Demethylation of **10** gave **11** as a multicaulin analogue, and then oxidation of **11** gave **12** as a miltirone analogue.

Compounds **9–12** were tested for their *in vitro* antimycobacterial activity against *M. smegmatis*, *M. bovis*, *M. szulgai*, *M. gastri*, *M. simiae*, and *M. tuberculosis* (H37Ra). The preliminary studies revealed that multicaulin analogues (**9**, **10** and **11**) were able to impair the growth of mycobacterial strains. Multicaulin analogues exhibited a considerable antimycobacterial activity; interestingly compound **11** showed a wider activity than the other compounds. The compound **11** showed higher or equal activity (except for *M. smegmatis* and *M. bovis*) than **9**, **10** and **12**.

MIC value of the most active compound (**11**) determined against the *M. tuberculosis* (H37Ra) was 8.26 μm when measured under *in vitro* condition. Compound **11** was also found to be equally effective against *M. bovis* with **9**, and **12**; against *M. szulgai* with **10** and **12**.

Compound **9** was totally inactive against all mycobacteria strains utilised. None of the compounds reported here was appreciably active against the *M. smegmatis* ATCC 14468 and *M. bovis* ATCC 35734.

Ulubelen et al. reported the activities of the compounds **5**–**8** against *M. tuberculosis* strain (H37Rv) with MIC values **6** (0.46 μg/ml), **8** (1.2 μg/ml), **7** (2.0 μg/ml) and **5** (5.6 μg/ml). Thus, 2-isopropylphenanthrene-3-ol structured compound **6** showed the strongest activity. Interestingly, among our synthesised compounds **9**–**12** the best biological activity was shown by 2-isopropylphenantherene-3-ol (**11**). These results imply that strong antituberculosis agents can be developed based on the structure of 2-isopropylphenantherene-3-ol (**11**).

In conclusion, we showed a new example of Pschorr reaction to synthesise multicaulin and miltirone analogues. Biological evaluation of the compounds suggests encouraging results for developing antituberculosis drugs based on phenanthrene derivatives.

## Supplementary Material

IENZ_1337758_Supplementary_Material.pdf
