# Supplementary material for: Syntheses and evaluation of multicaulin and miltirone-like compounds as antituberculosis agents
Source: J Enzyme Inhib Med Chem. 2017 Jun 29;32(1):878–84. doi: 10.1080/14756366.2017.1337758 (PMC6445210; doi:10.1080/14756366.2017.1337758)
Supplement: IENZ_1337758_Supplementary_Material.pdf [file IENZ_A_1337758_SM5053.pdf]

SUPPORTING INFORMATION

Syntheses and evaluation of multicaulin and miltirone-like compounds as antituberculosis agents

Serdar Burmaoğlu[a,b], Hatice Seçinti[a], Erkan Mozioğlu[c], Ahmet C. Gören[c], Ramazan Altundaş[a], Hasan Seçen[a]

[a]Department of Chemistry, Faculty of Science, Ataturk University, Erzurum, Turkey

[b]Tercan Vocational High School, Erzincan University, Erzincan, Turkey

[c]Chemistry Group Laboratories, TÜBİTAK, UME, Gebze-Kocaeli, Turkey

*Corresponding author: Tercan Vocational High School, Erzincan University, 24800, Erzincan, Turkey; Tel/Fax: +90-446-441-3627, +90-446-441-3672; e-mail: sburmaoglu@erzincan.edu.tr

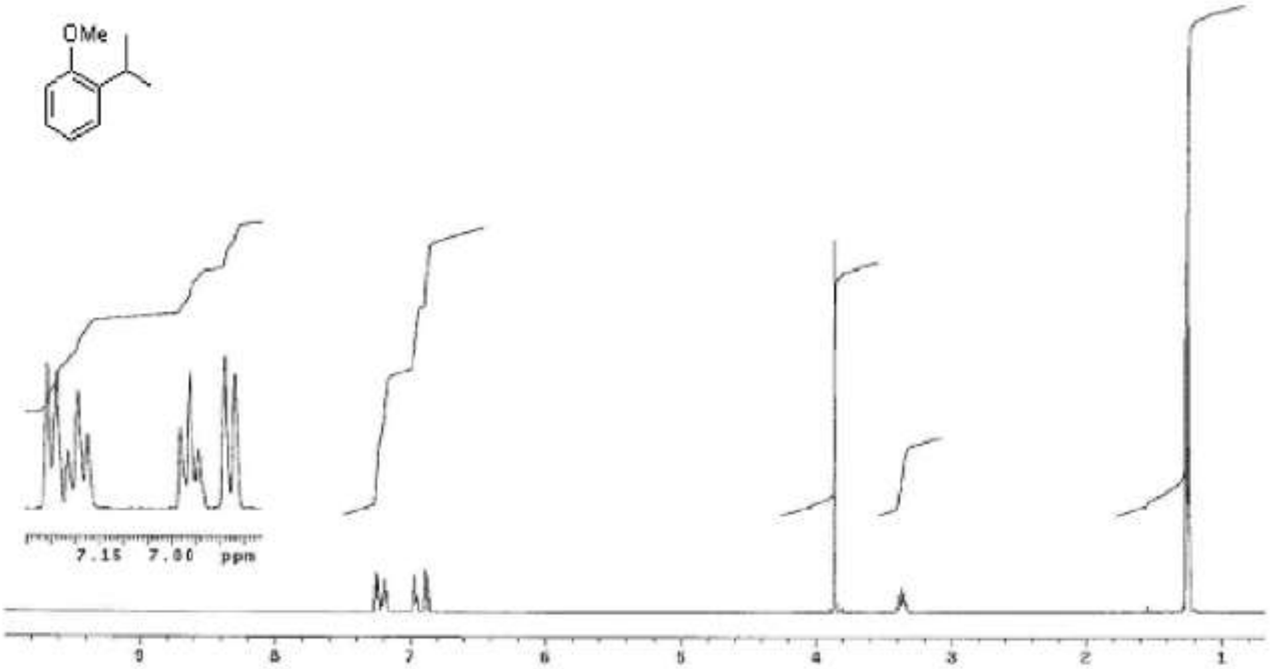

2-isopropylanisole **(18)** 400 MHz ^{1}H NMR Spectrum ($CDCl_3$): δ 7.35 (dd, 1 H, H-3, $J_{3,4}$ = 7.5 Hz, $J_{3,5}$ = 1.7 Hz); 7.29 (dt, 1 H, H-5, $J_{5,6}$ = $J_{4,5}$ = 8.0 Hz, $J_{3,5}$ = 1.7 Hz); 7.06 (dt, 1 H, H-4, $J_{4,5}$ = $J_{5,6}$ = 8.0 Hz, $J_{4,6}$ = 1.1 Hz), 6.96 (dd, 1 H, H-6, $J_{5,6}$ = 8.0 Hz, $J_{4,6}$ = 1.1 Hz); 3.93 (s, 3H, OCH_3); 3.50 (septet, 1H, C<u>H</u>Me_2, J = 7.0 Hz); 1.36 (d, 6H, CH<u>Me_2</u>, J= 7.0 Hz).

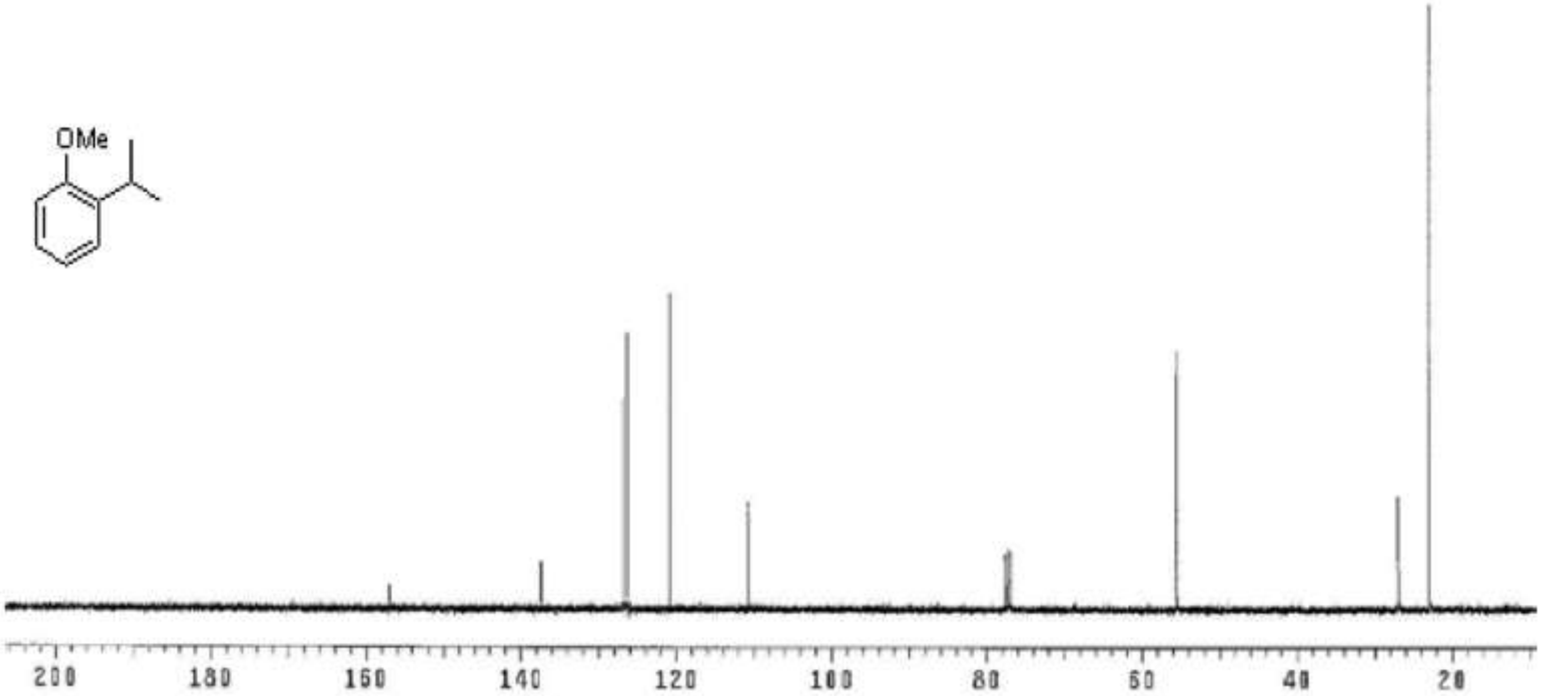

2-isopropylanisole **(18)** 100 MHz ^{13}C NMR Spectrum ($CDCl_3$): δ 156.7 (C-1); 136.9 (C-2); 126.5 (C-3); 125.9 (C-5); 120.5 (C-4); 110.2 (C-6); 55.2 (OMe); 26.6 (<u>C</u>HMe$_2$); 22.6 (CH<u>Me$_2$</u>).

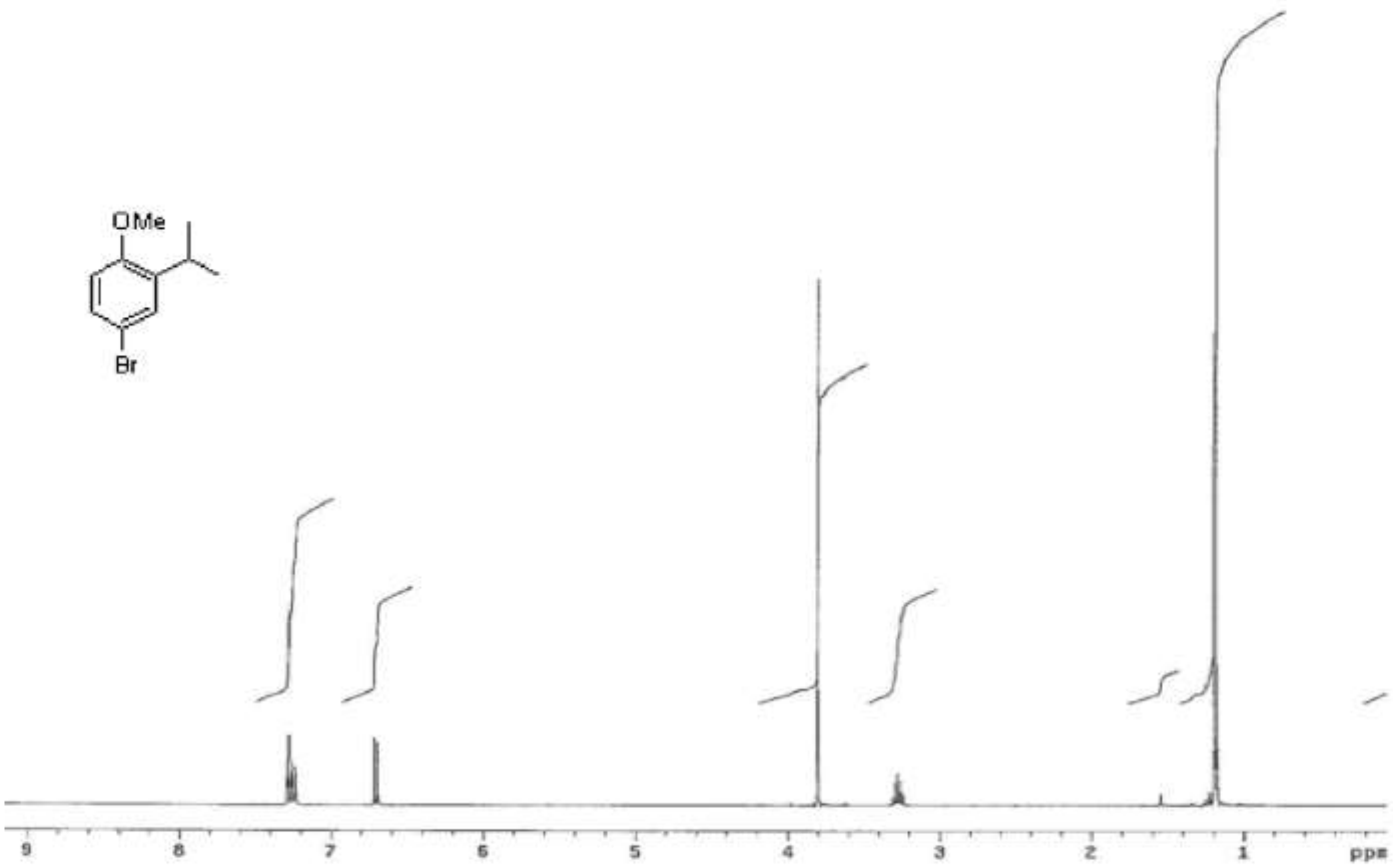

4-bromo-2-isopropylanisole **(19)** 400 MHz ^{1}H-NMR Spectrum ($CDCl_3$): δ 7.34 (d, 1 H, H-3, $J_{3,5}$ = 2.4 Hz); 7.29 (dd, 1H, H-5, $J_{5,6}$ = 8.5 Hz, $J_{3,5}$ = 2.4 Hz); 6.73 (d, 1H, H-6, $J_{5,6}$ = 8.5 Hz); 3.83 (s, 3H, OCH_3); 3.33 (septet, 1H, C$\underline{H}$$Me_2$, J = 6.9 Hz); 1.24 (d, 6H, CH$\underline{Me_2}$, J = 6.9 Hz).

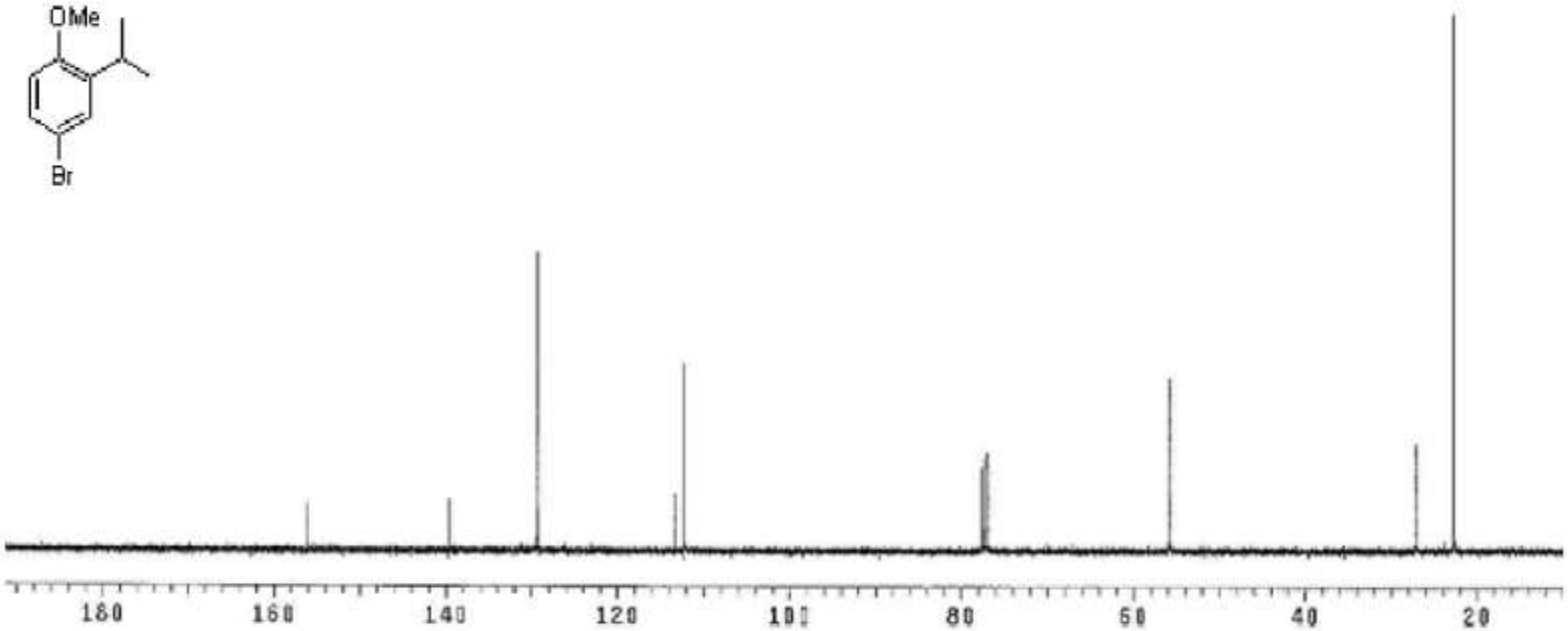

4-bromo-2-isopropylanisole **(19)** 100 MHz ^{13}C-NMR Spectrum ($CDCl_3$): δ 155.9 (C-1); 139.4 (C-2); 129.1 (C-3); 129.0 (C-5); 113.0 (C-4); 112.0 (C-6); 55.4 (OCH_3); 26.8 ($\underline{C}HMe_2$); 22.4 ($CH\underline{Me}_2$).

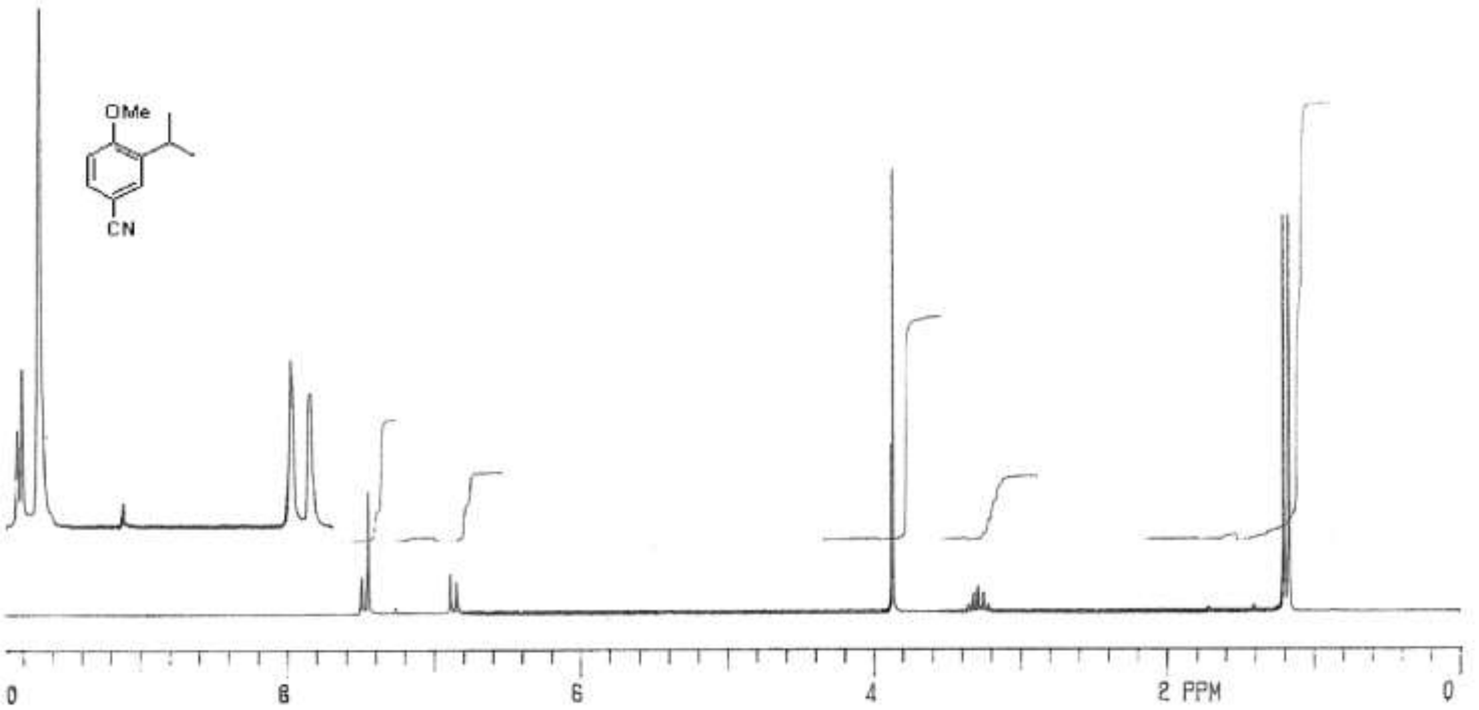

3-isopropyl-4-methoxybenzonitrile (**20**) 200 MHz ^{1}H-NMR Spectrum ($CDCl_3$) δ 7.46 (dd, 1H, H-6, $J_{5,6}$=8.6 Hz, $J_{2,6}$=2.1 Hz); 7.45 (d, 1H, H-2, $J_{2,6}$=2.1 Hz); 6.87 (d, 1H, H-5, $J_{5,6}$=8.6 Hz) 3.87 (s, 3H, OCH_3); 3.29 (septet, 1H, C<u>H</u>Me_2, J=6.8 Hz); 1.18 (d, 6H, CH<u>Me$_2$</u>, J=6.8 Hz).

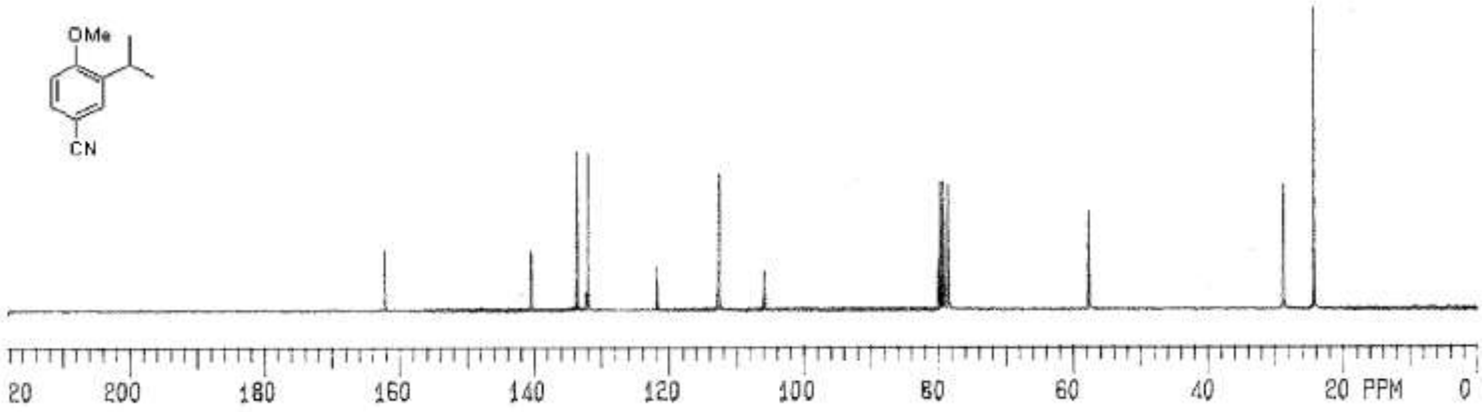

3-isopropyl-4-methoxybenzonitrile (**20**) 50 MHz ^{13}C-NMR Spectrum ($CDCl_3$) δ 159.1 (C-4); 138.3 (C-3); 131.4 (C-2 or C-6); 129.9 (C-2 or C-6); 119.6 (CN); 110.5 (C-5); 103.7 (C-1); 55.5 (OCH_3); 26.6 (<u>C</u>HMe$_2$); 22.1 (CH<u>Me$_2$</u>,).

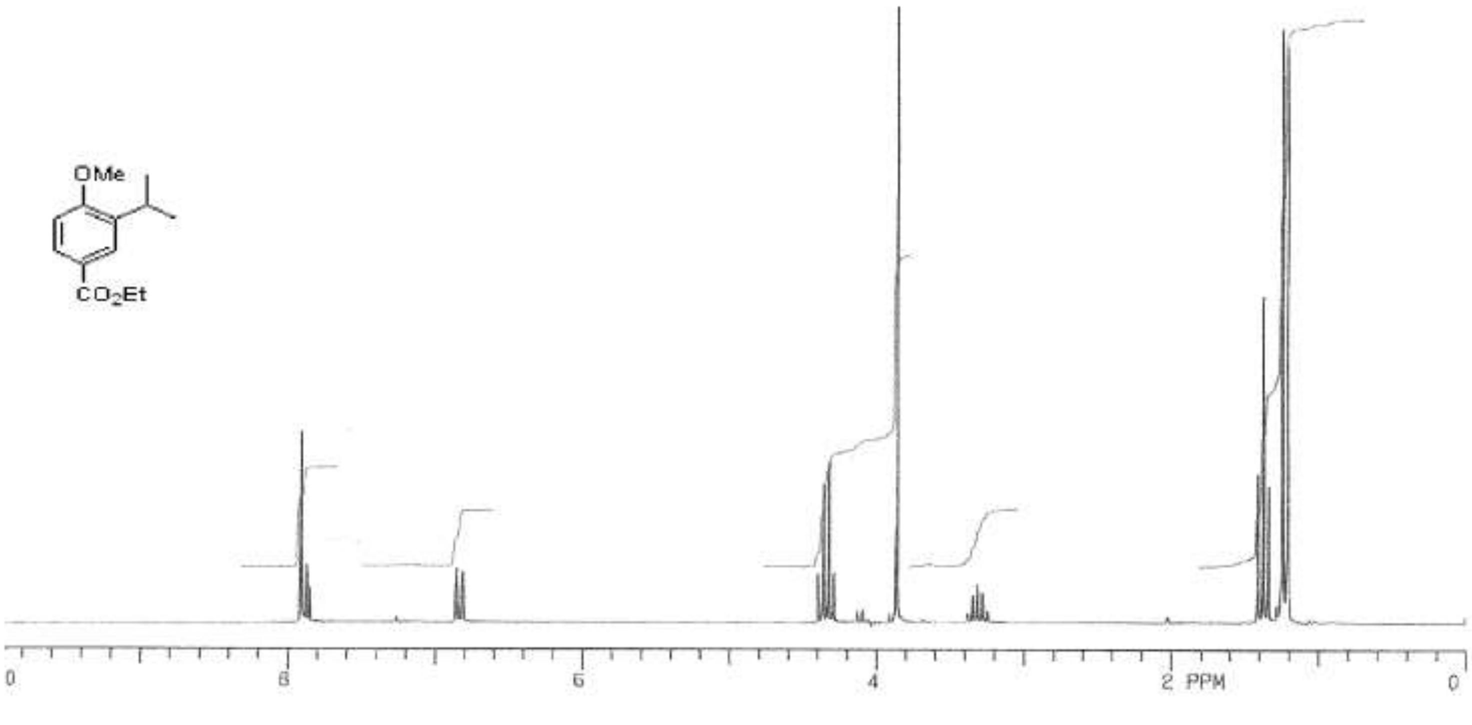

Ethyl 3-isopropyl–4-methoxybenzoate (**21**) 200 MHz ^{1}H-NMR Spectrum ($CDCl_3$) δ 7.90 (s, 1H, H-2); 7.88 (d, 1H, H-6, $J_{5,6}$=8.3 Hz); 6.82 (d, 1H, H-5, $J_{5,6}$=8.3 Hz); 4.34 (q, 2H, O$\underline{CH_2}$$CH_3$, J=7.0 Hz); 3.85 (s, 3H, OCH_3); 3.31 (septet, 1H, $\underline{CH}Me_2$, J=7.0 Hz); 1.37 (t, 3H, $OCH_2\underline{CH_3}$, J=7.0 Hz); 1.22 (d, 6H, $CH\underline{Me_2}$, J=7.0 Hz).

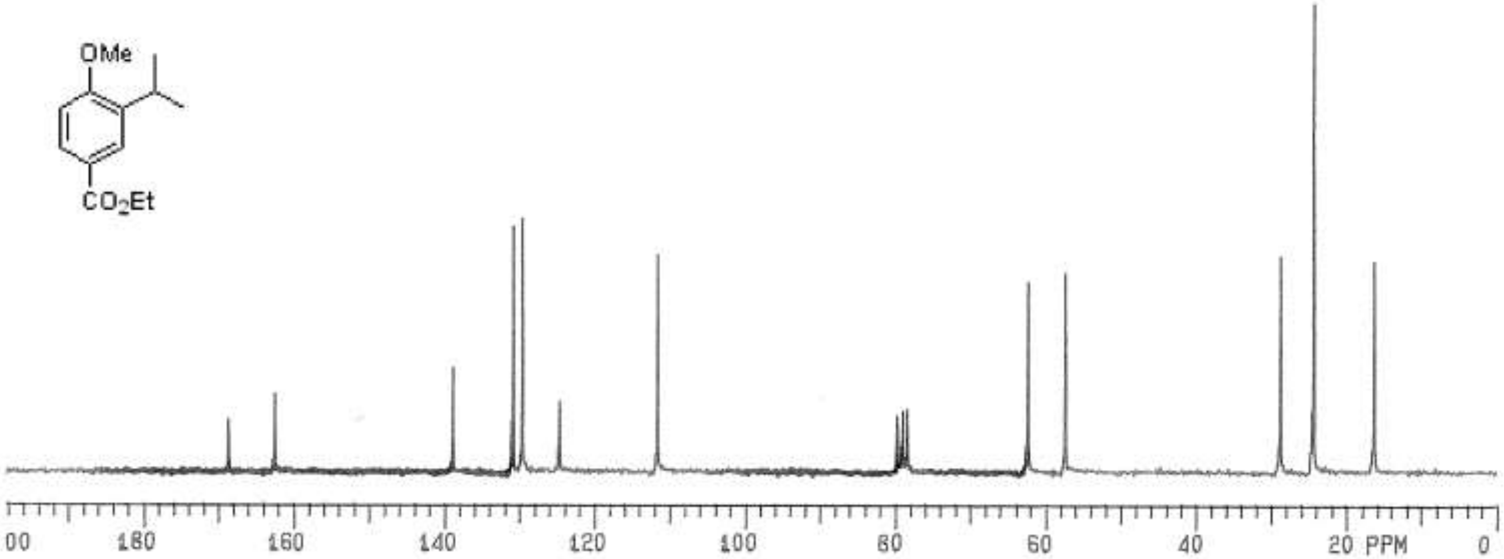

Ethyl 3-isopropyl–4-methoxybenzoate (**21)** 50 MHz ^{13}C-NMR Spectrum ($CDCl_3$) δ 166.5 (ester karbonili); 160.5 (C-4); 136.8 ((C-1); 128.8 (C-6); 127.6 (C-2); 122.6 (C-3); 109.5 (C-5); 60.3 (O<u>C</u>H_2CH_3); 55.3 (OCH_3); 26.7 (<u>C</u>HMe$_2$); 22.2 (CH<u>Me$_2$</u>); 14.2 (OCH_2<u>C</u>H_3).

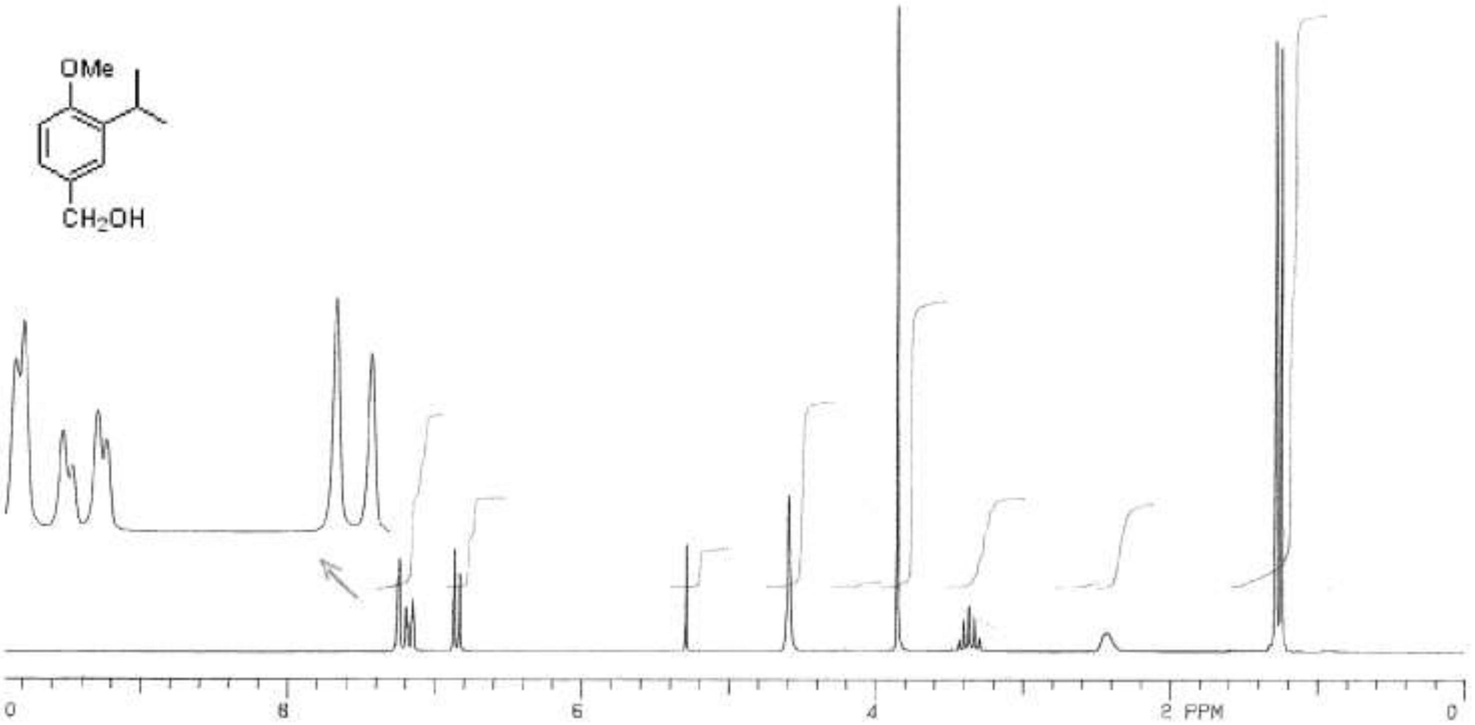

3-isopropyl–4-methoxybenzylalcohol (**22**) 200 MHz ^{1}H-NMR Spectrum ($CDCl_3$) δ 7.24 (d, 1H, H-2, $J_{2,6}$=2.0 Hz); 7.18 (dd, 1H, H-6, $J_{5,6}$=8.1 Hz, $J_{2,6}$=2.0 Hz); 6.84 (d, 1H, H-5, $J_{5,6}$=8.1 Hz); 4.59 (s, 2H, C$\underline{H}_2$OH); 3.85 (s, 3H, OCH_3); 3.36 (septet, 1H, C$\underline{H}$Me$_2$, J=7.0 Hz); 2.43 (bs, 1H, OH); 1.26 (d, 6H, CH$\underline{Me}_2$, J=7.0 Hz).

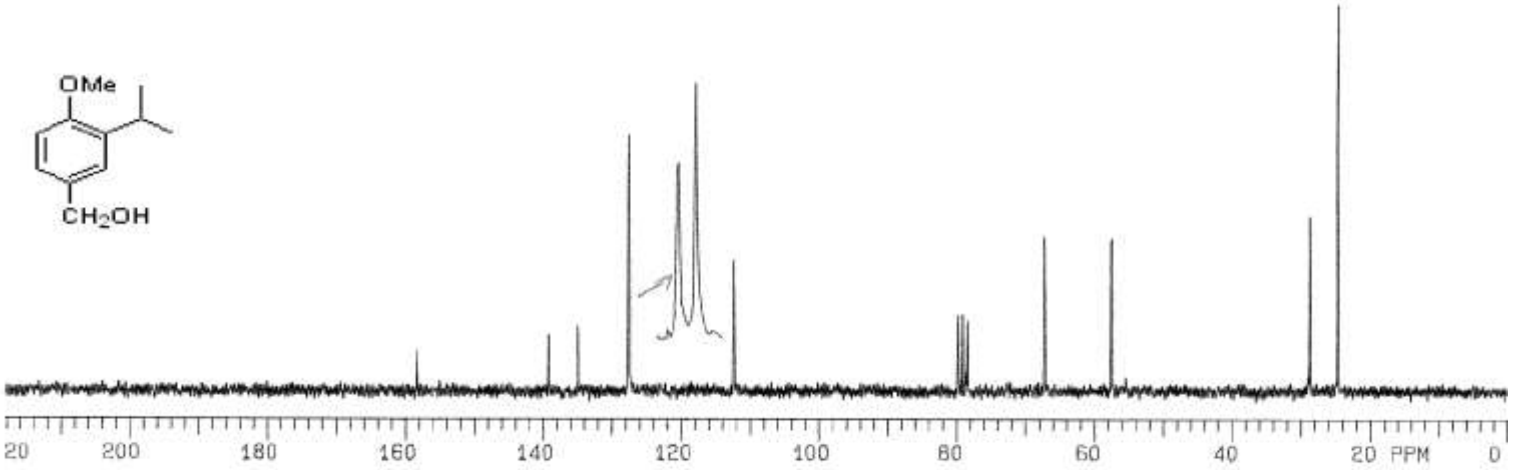

3-isopropyl–4-methoxybenzylalcohol (**22**) 50 MHz ^{13}C-NMR Spectrum ($CDCl_3$) δ 156.2 (C-4); 137.0 (C-3); 132.8 (C-1); 125.4 (C-2 or C-6); 125.2 (C-2 or C-6); 110.2 (C-5); 65.0 ($\underline{C}H_2OH$); 55.3 (OCH_3); 26.6 ($\underline{C}HMe_2$); 22.5 ($CH\underline{Me_2}$).

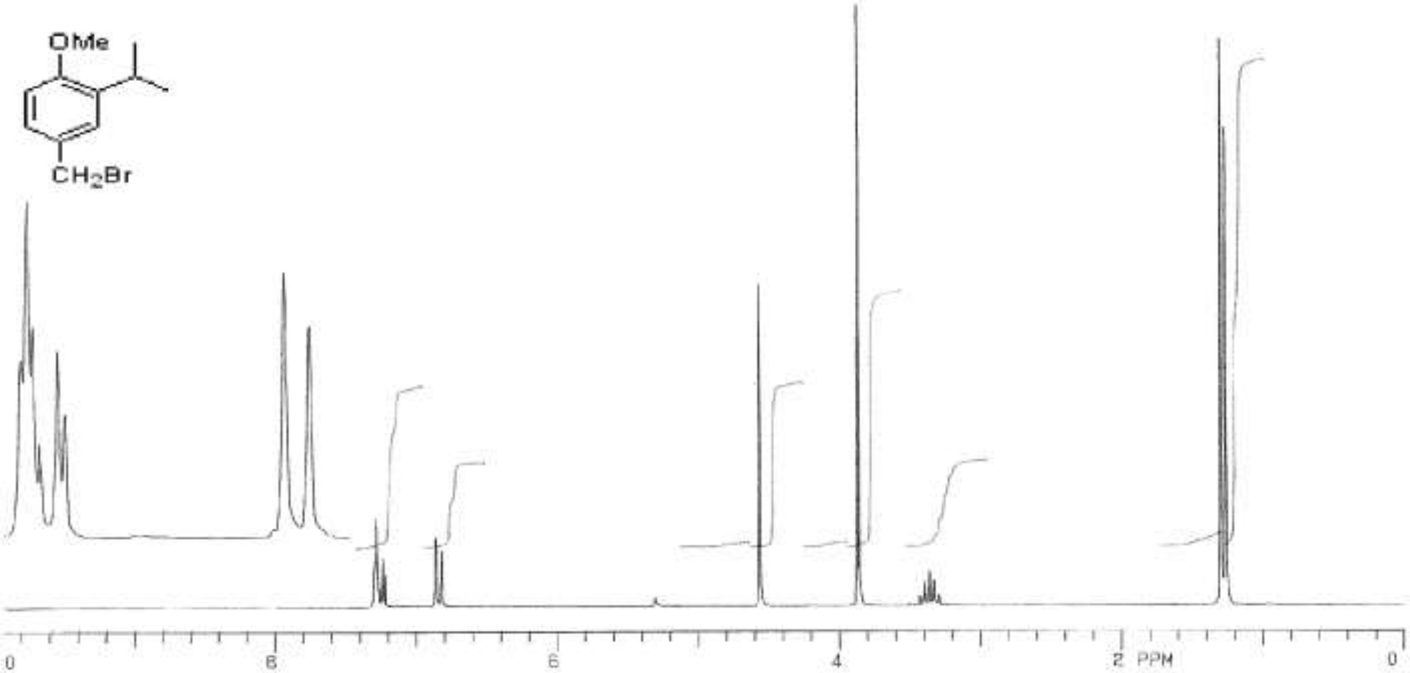

3-isopropyl–4-methoxybenzylbromide (**23**) 200 MHz ^{1}H-NMR Spectrum ($CDCl_3$) δ 7.29 (d, 1H, H-2, $J_{2,3}$=2.2 Hz); 7.26 (dd, 1H, H-6, $J_{5,6}$=8.2 Hz, $J_{2,3}$=2.2 Hz); 6.84 (d, 1H, H-5, $J_{5,6}$=8.2 Hz); 4.56 (s, 2H, C$\underline{H_2}$Br); 3.86 (s, 3H, OCH_3); 3.36 (septet, 1H, C<u>H</u>Me_2, J=6.9 Hz); 1.28 (d, 6H, CH$\underline{Me_2}$, J=6.9 Hz).

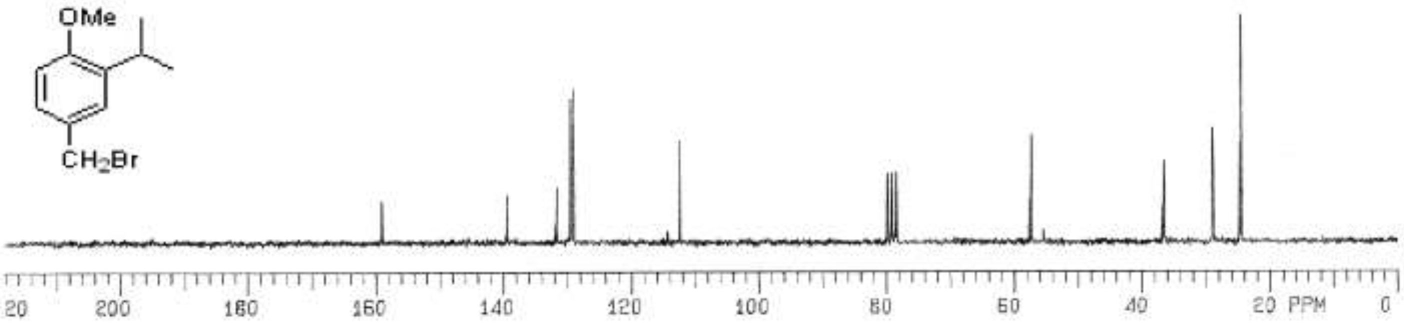

3-isopropyl–4-methoxybenzylbromide (**23**) 50 MHz ^{13}C-NMR Spectrum ($CDCl_3$) δ 156.9 (C-4); 137.4 (C-3); 129.7 (C-1); 127.4 (C-2 or C-6); 127.1 (C-2 or C-6); 110.4 (C-5); 55.4 (OCH_3); 34.5 (CH_2Br); 26.8 ($\underline{C}HMe_2$); 22.5 ($CH\underline{Me}_2$).

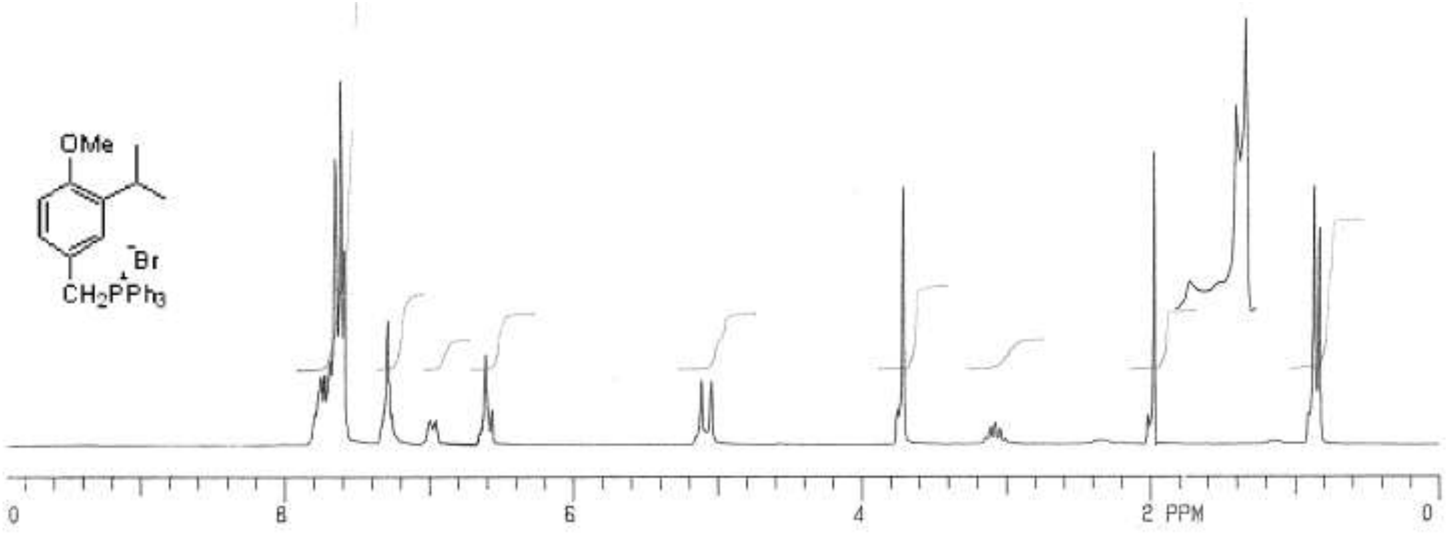

(3-Isopropyl-4-methoxybenzyl)triphenylphosphoniumbromide **(16)** 200 MHz ^{1}H-NMR Spectrum ($CDCl_3$)

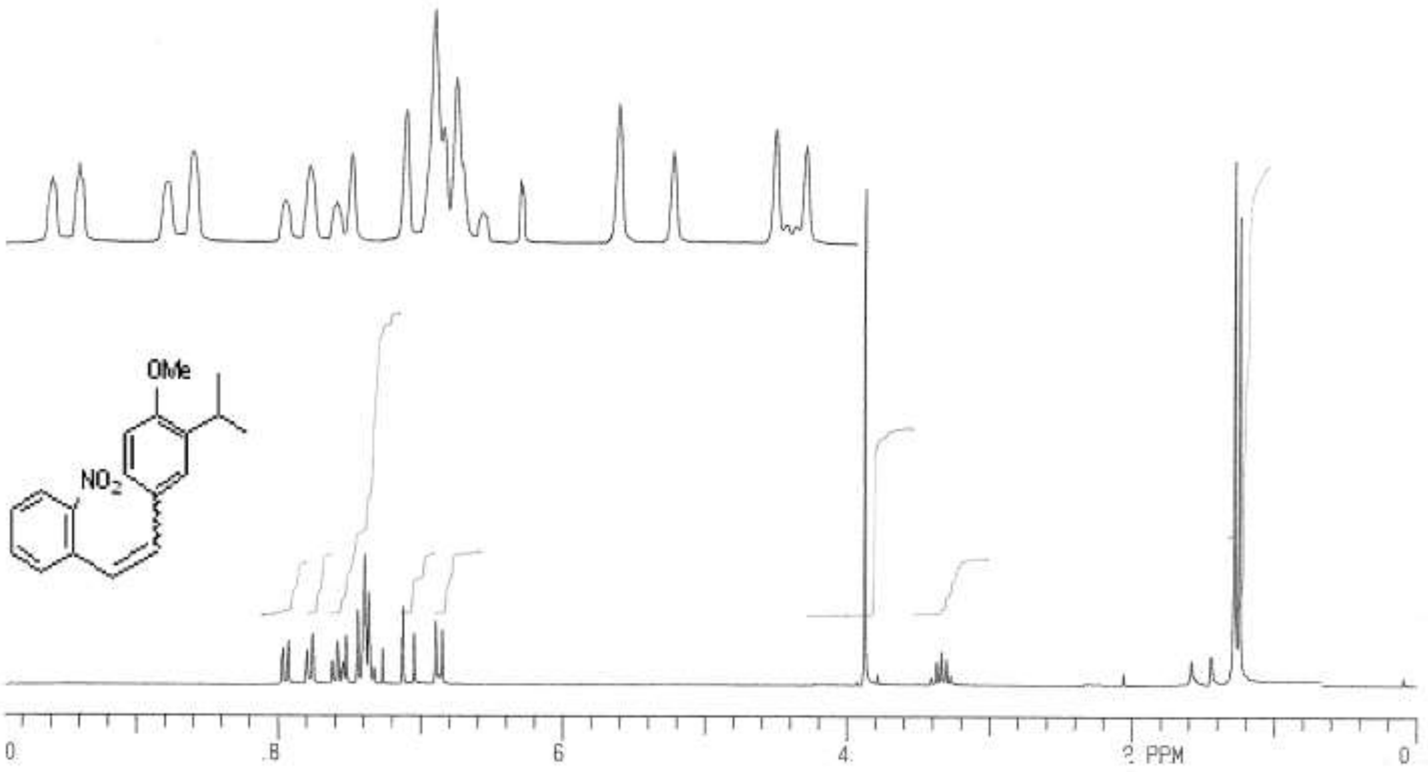

(*E*)-1-(3-isopropyl-4-methoxyphenyl)-2-(2-nitrophenyl)ethene (**14**) 400 MHz ^{1}H-NMR Spectrum ($CDCl_3$) δ 7.94 (dd, 1H, H-3’’[a], $J_{3'',4''}$=8.1 Hz, $J_{3'',5''}$=1.5 Hz); 7.76 (dd, 1H, H-6’’[a], $J_{5'',6''}$=8.1 Hz, $J_{3'',5''}$=1.5 Hz); 7.57 (bt, 1H, H-5’’[b], $J_{5'',6''}$=$J_{4'',5''}$=8.1 Hz); 7.46 (d, 1H, H-2, $J_{1,2}$=16.1 Hz); 7.38 (s, 1H, H-2’); 7.37 (d, 1H, H-6’, $J_{5',6'}$=9.1 Hz); 7.36 (bt, 1H, H-4’’[b], $J_{4'',5''}$=$J_{3'',4''}$=8.1 Hz); 7.08 (d, 1H, H-1, $J_{1,2}$=16.1 Hz); 6.86 (d, 1H, H-5’, $J_{5',6'}$=9.1 Hz); 3.86 (s, 3H, OCH_3); 3.33 (septet, 1H, C<u>H</u>Me_2, J=6.6 Hz); 1.24 (d, 6H, CH<u>Me_2</u>, J=6.6 Hz).

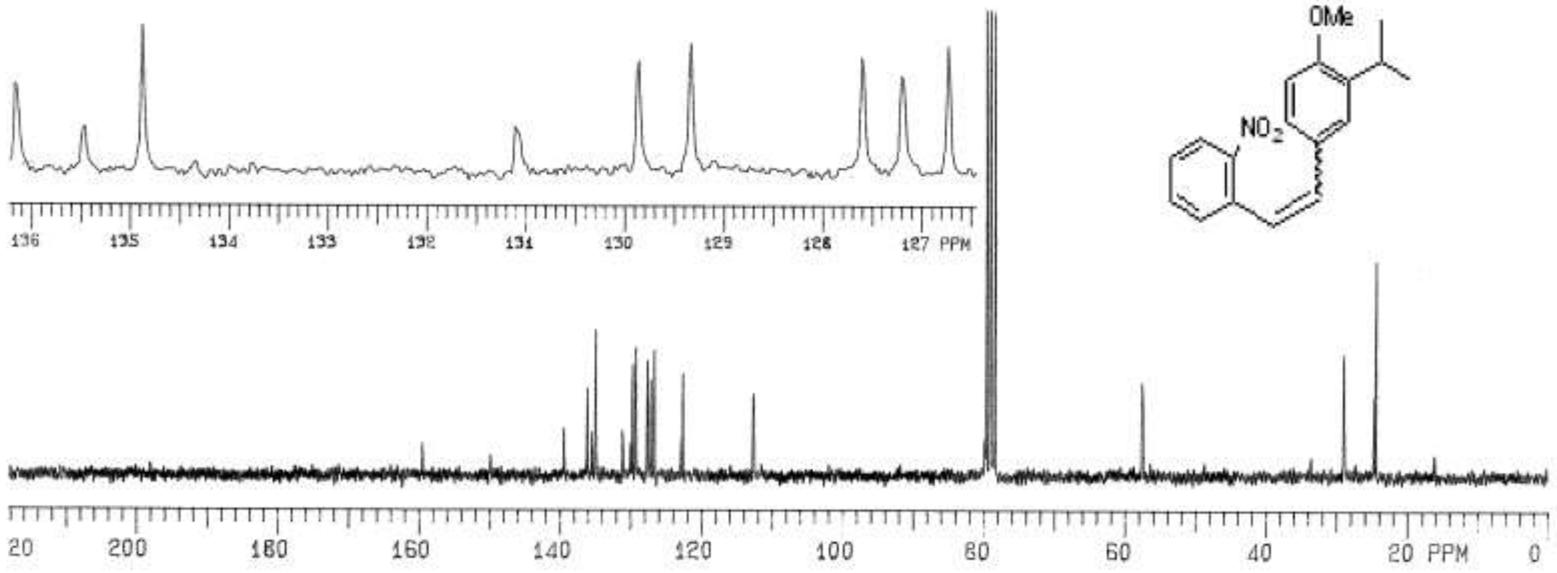

(*E*)-isomer of 1-(3-isopropyl-4-methoxyphenyl)-2-(2-nitrophenyl)etene **(14)** (400 MHz, $CDCl_3$) δ 7.94 (dd, 1H, H-3’’[a], J = 8.1 Hz, J = 1.5 Hz); 7.76 (dd, 1H, H-6’’[a], J = 8.1 Hz, J = 1.5 Hz); 7.57 (bt, 1H, H-5’’[b], J = 8.1 Hz); 7.46 (d, 1H, H-2, J = 16.1 Hz); 7.38 (s, 1H, H-2’); 7.37 (d, 1H, H-6’, J = 9.1 Hz); 7.36 (bt, 1H, H-4’’[b], J = 8.1 Hz); 7.08 (d, 1H, H-1, J = 16.1 Hz); 6.86 (d, 1H, H-5’, J = 9.1 Hz); 3.86 (s, 3H, OMe); 3.33 (septet, 1H, $CHMe_2$, J = 6.6 Hz); 1.24 (d, 6H, $CHMe_2$, J = 6.6 Hz).

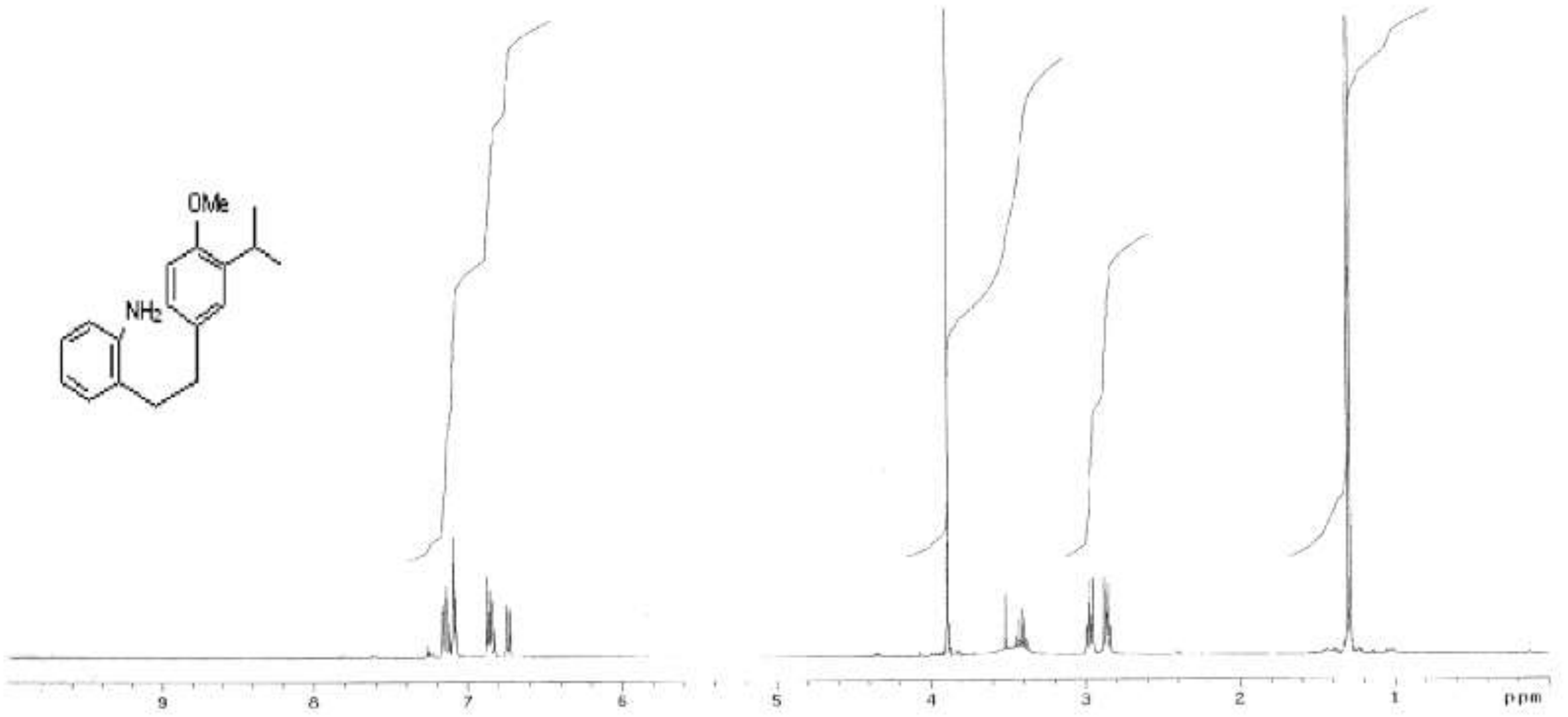

1-(3-isopropyl-4-methoxyphenyl)-2-(2-aminophenyl)ethane (**13**) 400 MHz ^{1}H-NMR Spectrum ($CDCl_3$) δ 7.16-7.01 (m, 4H, H-4’’, H-6’’, H-2’, H-6’); 6.86 (d, 1H, H-5’, $J_{5',6'}$=9.0 Hz); 6.84 (dt, 1H, H-5’’, $J_{4'',5''}$=$J_{5'',6''}$=7.4 Hz, $J_{3'',6''}$=1.2 Hz); 6.74 (bd, 1H, H-3’’, $J_{3'',4''}$=7.8 Hz); 3.89 (s, 3H, OCH_3); 3.44 (bs, 2H, NH_2); 3.40 (septet, 1H, C<u>H</u>Me_2, J=7.0 Hz); 3.00-2.83 (m, 4H, A_2B_2 system, 2xH-1, 2xH-2); 1.29 (d, 6H, CH<u>Me</u>$_2$, J=7.0 Hz).

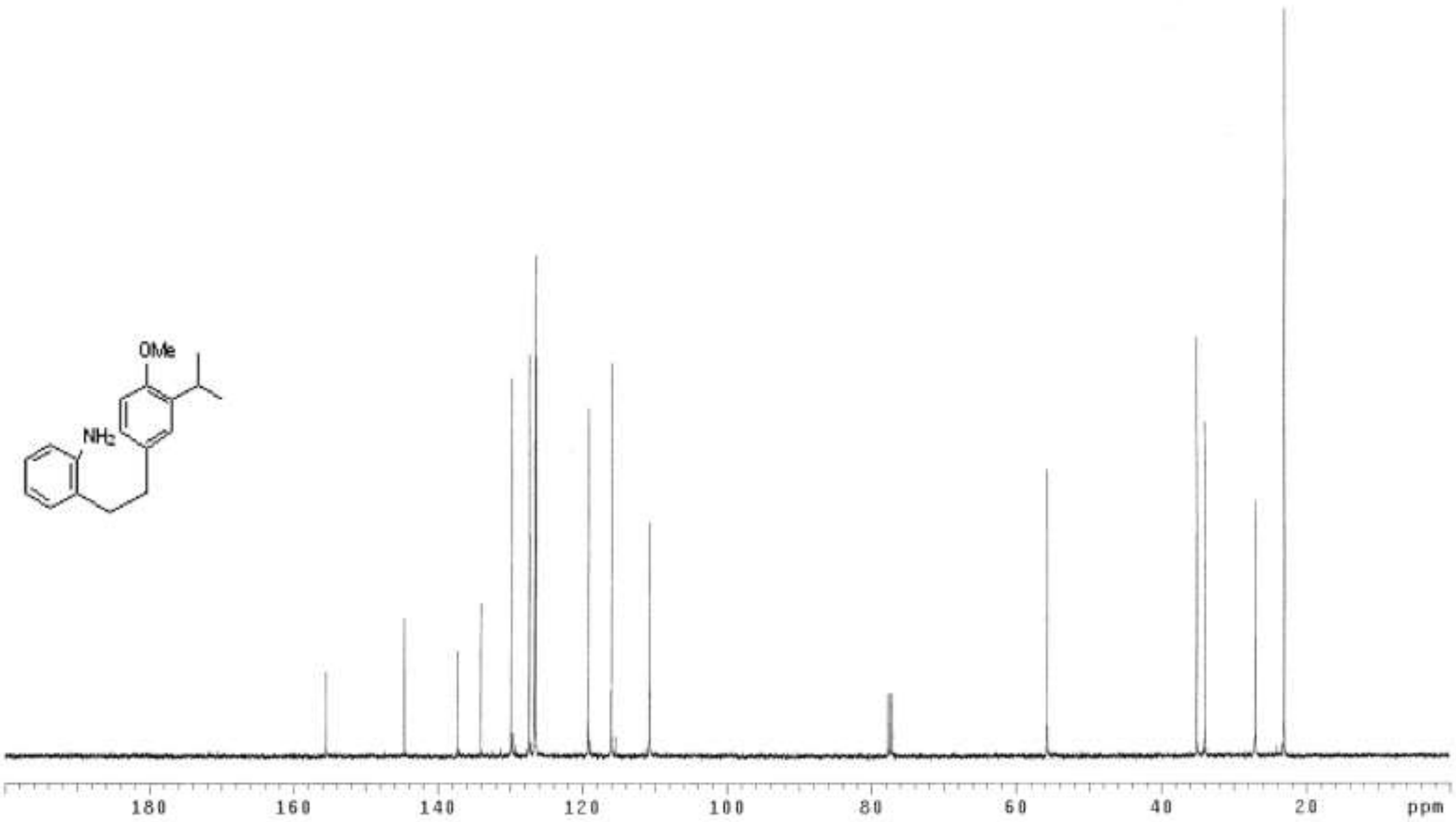

1-(3-isopropyl-4-methoxyphenyl)-2-(2-aminophenyl)ethane (**13**) 100 MHz ^{13}C-NMR Spectrum ($CDCl_3$) δ 155.1 (C-4’); 144.2 (C-2’’); 136.8 (s); 133.6 (s); 129.4 (d); 127.0 (d); 126.3 (s); 126.2 (d); 126.1 (d); 118.8 (d); 115.6 (d); 110.4 (d); 55.4 (OCH_3); 34.7 (C-1[a]); 33.6 (C-2[a]); 26.6 ($\underline{C}HMe_2$); 22.6 ($CH\underline{Me_2}$).

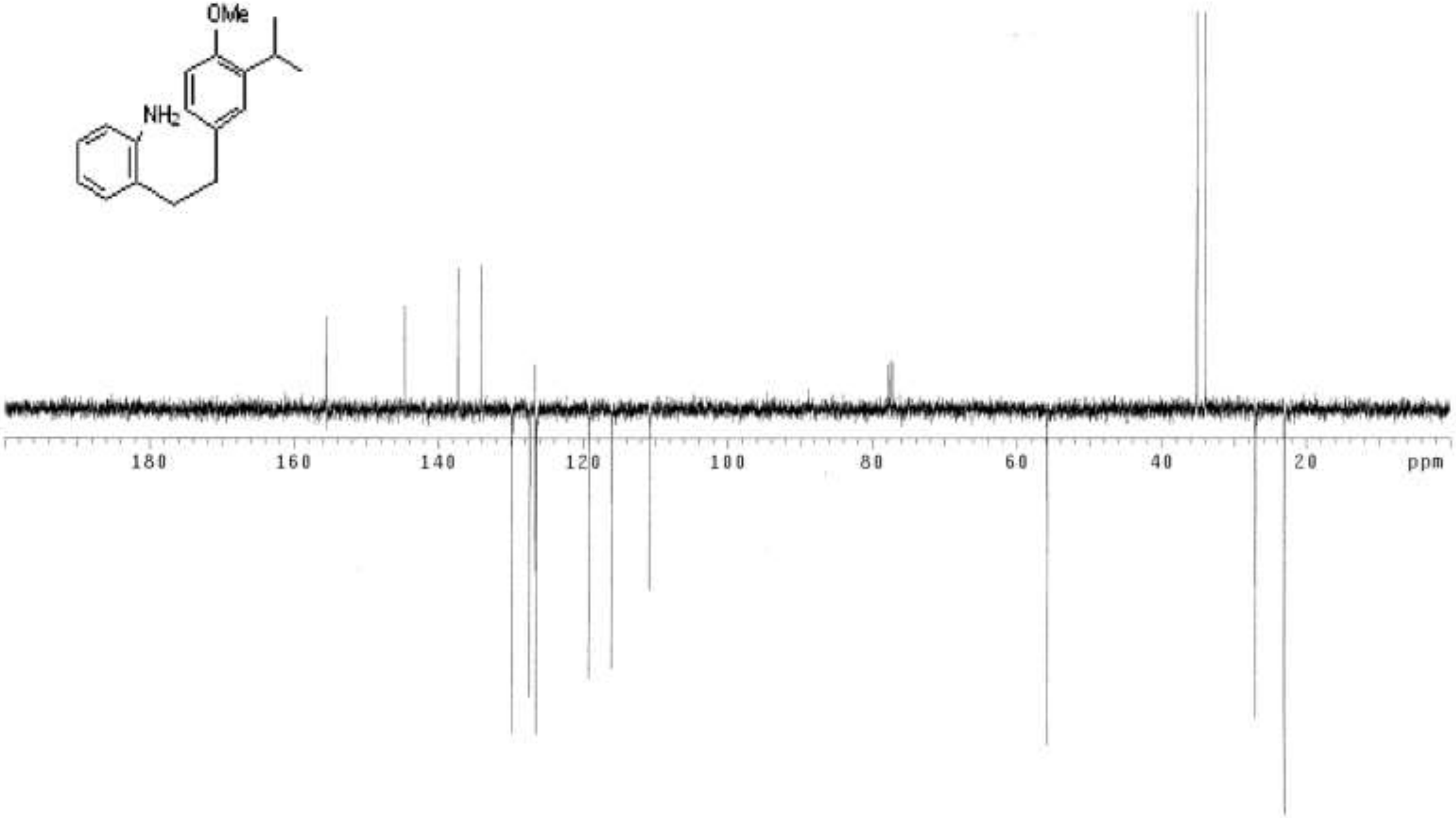

1-(3-isopropyl-4-methoxyphenyl)-2-(2-aminophenyl)ethane (**13**) 100 MHz APT Spectrum

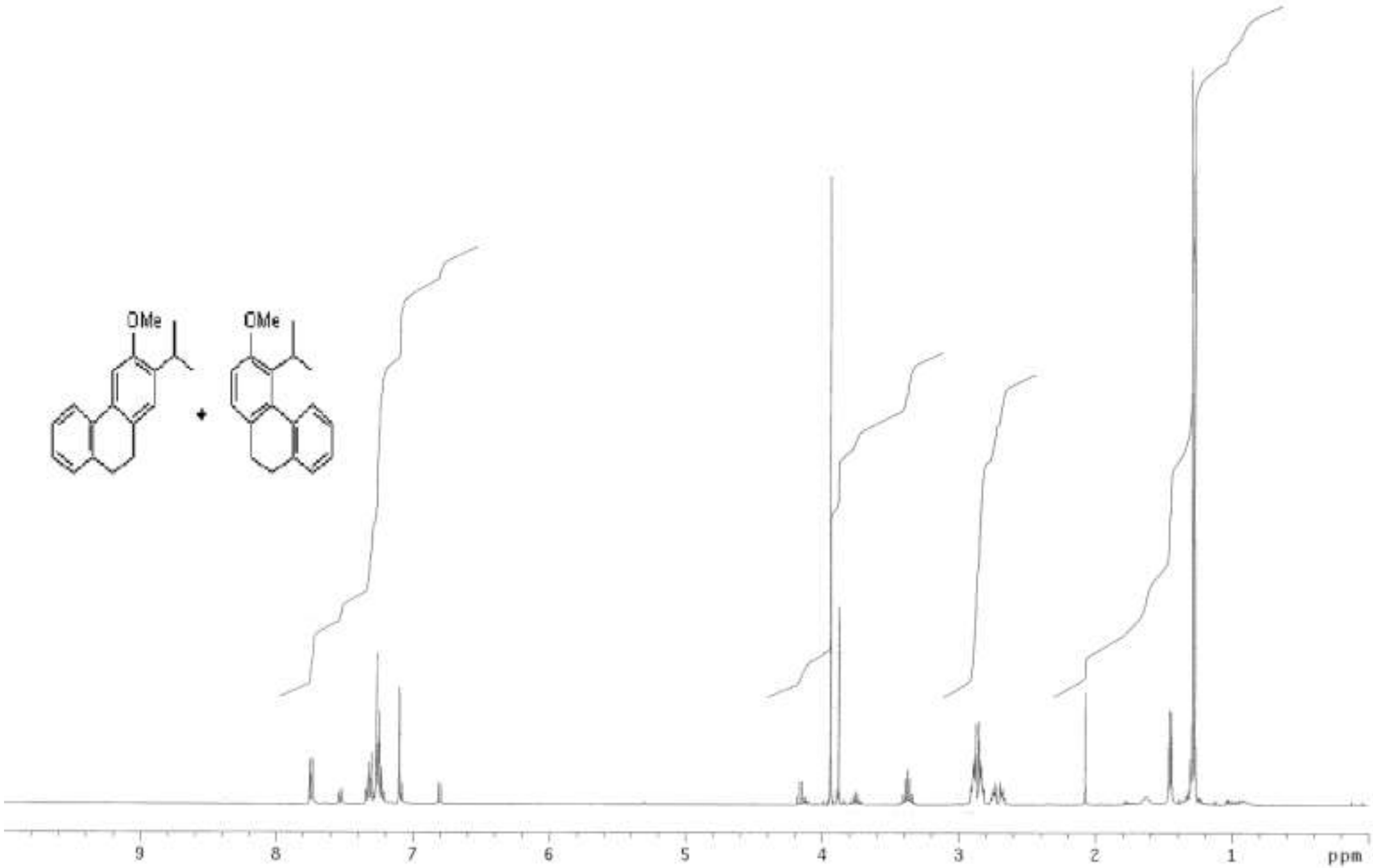

400 MHz ^{1}H-NMR Spectrum of mixture **9** and **9a** ($CDCl_3$)

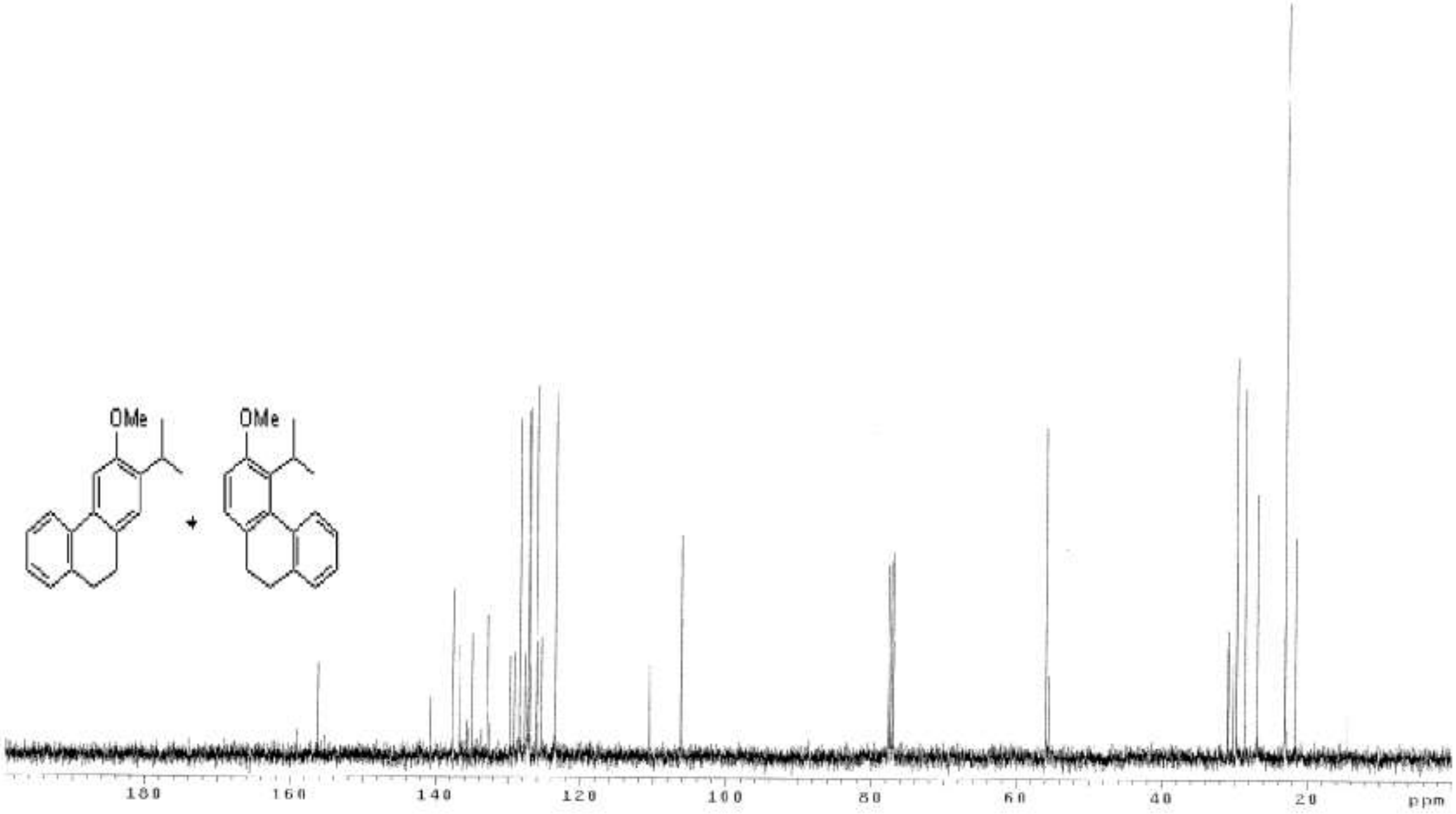

100 MHz ^{13}C-NMR Spectrum of mixture **9** and **9a** ($CDCl_3$)

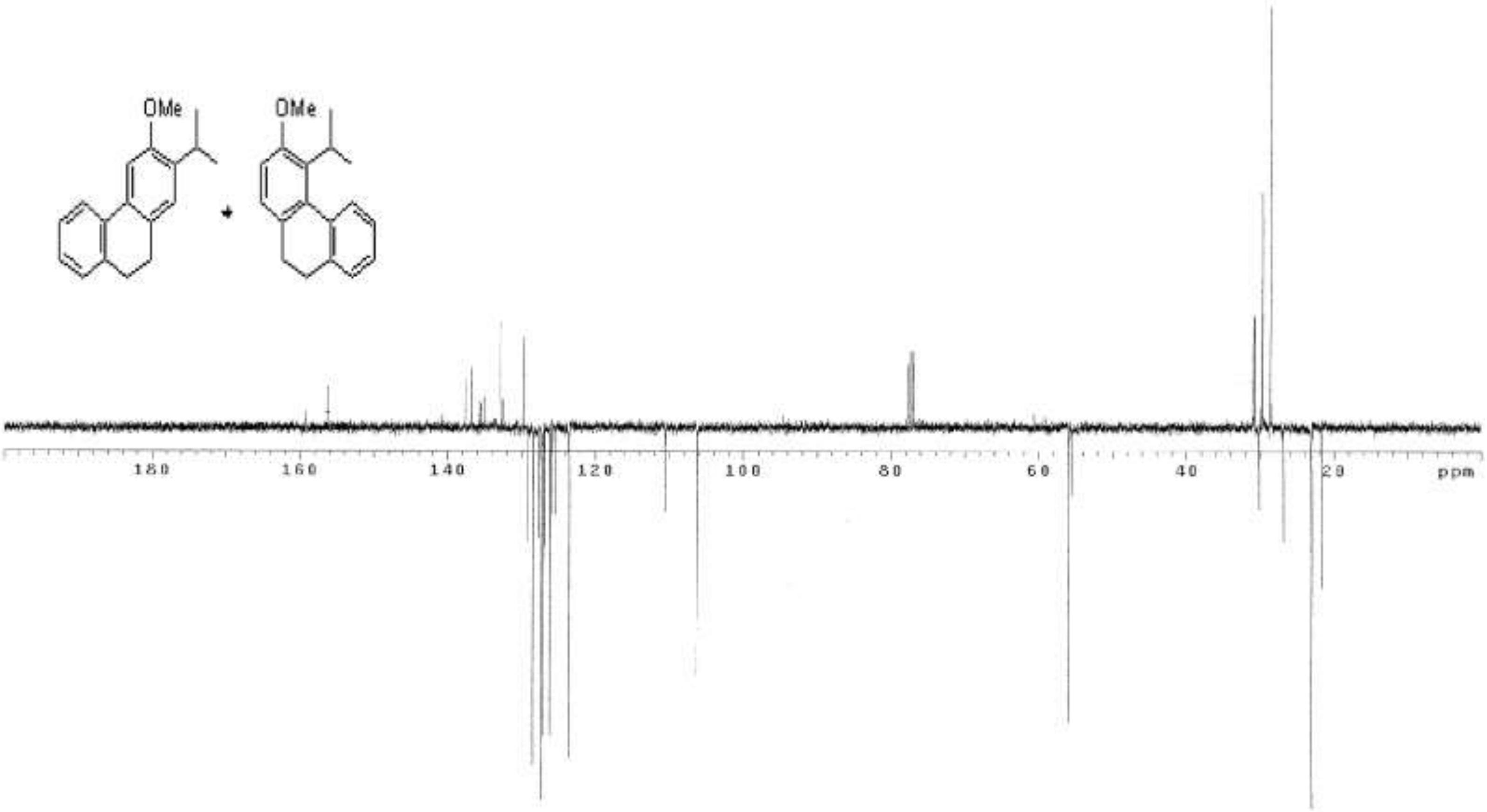

100 MHz APT Spectrum of mixture **9** and **9a** ($CDCl_3$)

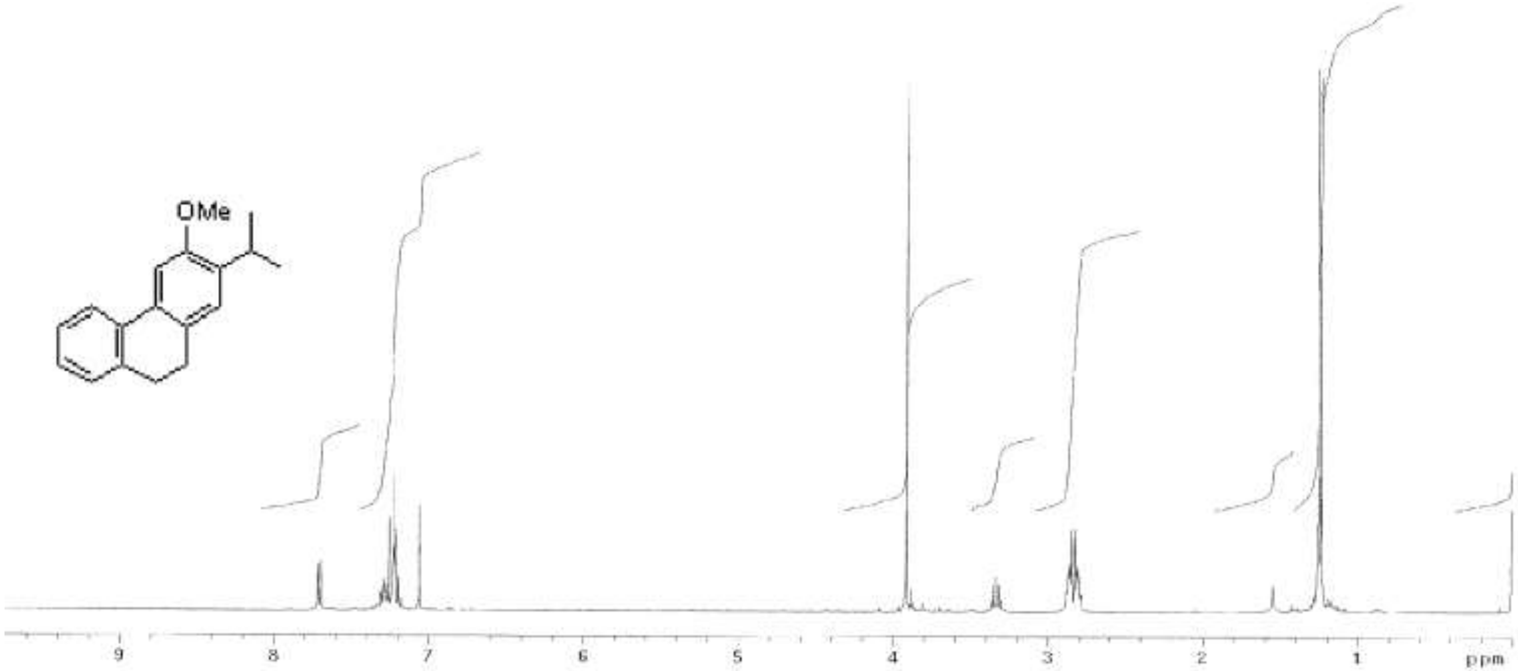

2-isopropyl-3-methoxy-9,10-dihydrophenanthrene (**9**) 400 MHz ^{1}H-NMR Spectrum ($CDCl_3$) δ 7.71 (bd, 1H, H-5, $J_{5,6}$=7.7 Hz); 7.29 (ddd, quasi dt, 1H, H-6, $J_{5,6}$=7.7 Hz, $J_{6,7}$=6.9 Hz, $J_{6,8}$=1.8 Hz); 7.23 (s, 1H, H-1); 7.22-7.18 (m, 2H, H-7 ve H-8); 7.01 (s, 1H, H-4); 3.91 (s, 3H, OCH_3); 3.33 (septet, 1H, $C\underline{H}Me_2$, J=7.0 Hz); 2.88-2.78 (m, A_2B_2 system, 4H, 2xH-9 ve 2xH-10); 1.24 (d, 6H, $CH\underline{Me_2}$, J=7.0 Hz).

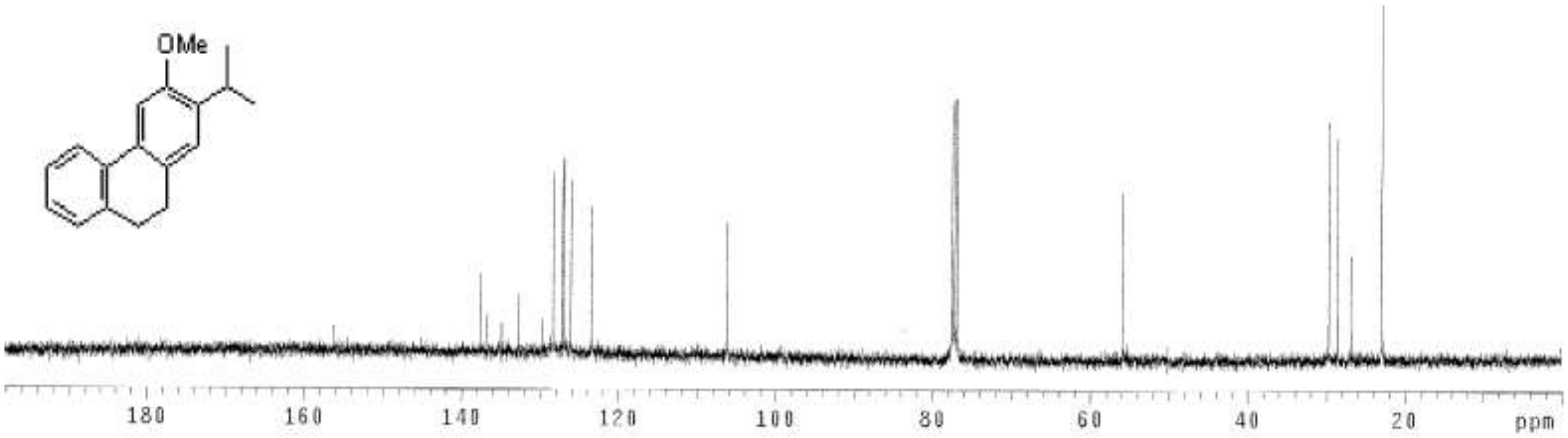

2-isopropyl-3-methoxy-9,10-dihydrophenanthrene (**9**) 100 MHz ^{13}C-NMR Spectrum ($CDCl_3$) δ 156.0 (C-5); 137.3 (s); 136.5 (s); 134.7 (s); 132.5 (s); 129.5 (s); 128.1 (d); 127.0 (d); 126.8 (d); 125.9 (d); 123.3 (d); 106.0 (d); 55.7 (OCH_3); 29.5 (C-9[a]); 28.3 (C-10[a]); 26.7 ($\underline{C}HMe_2$); 22.8 ($CH\underline{Me_2}$).

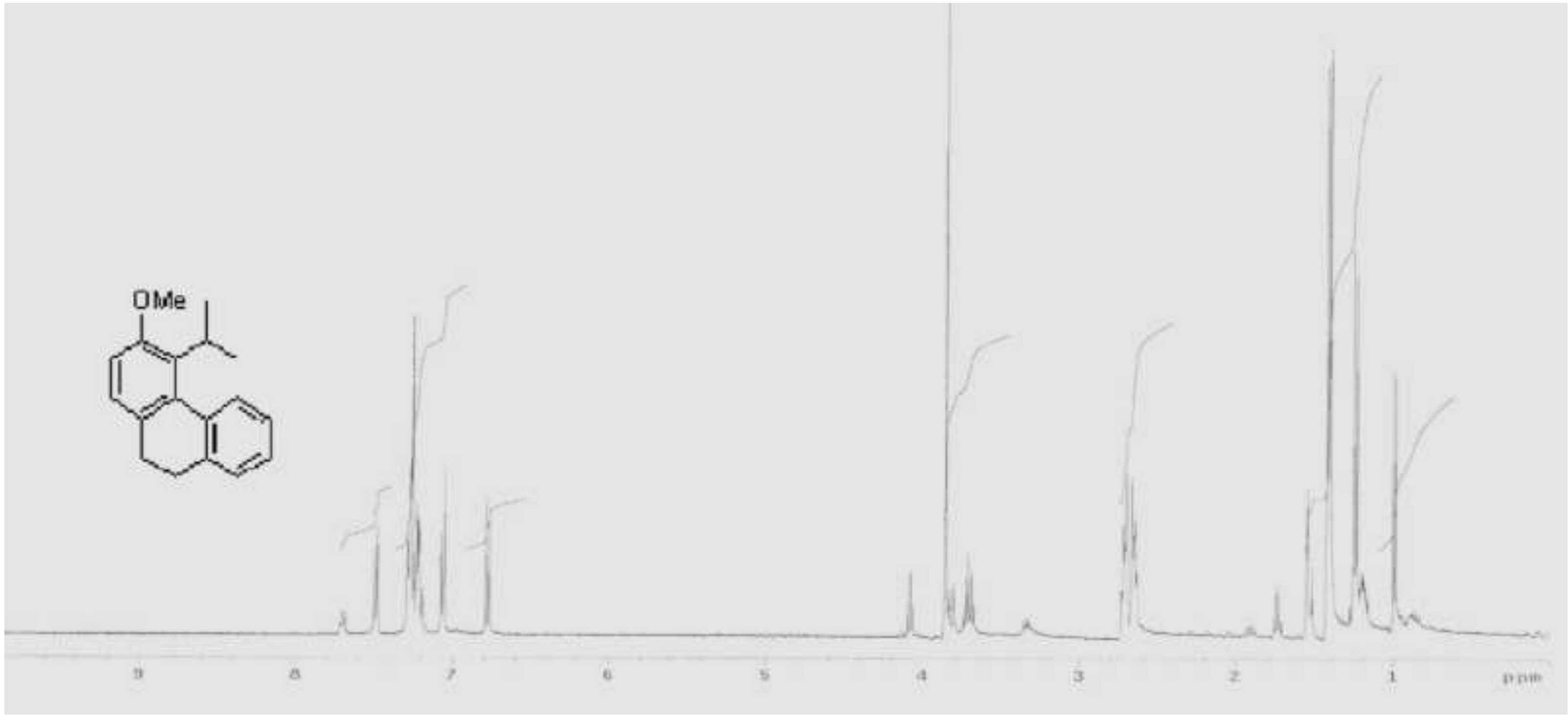

4-isopropyl-3-methoxy-9,10-dihydrophenanthrene (**9a**) 400 MHz ^{1}H-NMR Spectrum ($CDCl_3$) δ 7.49 (bd, 1H, H-5, $J_{5,6}$=7.7 Hz); 7.29-7.19 (m, 3H, H-6, H-7, H-8); 7.06 (d, 1H, H-1, $J_{1,2}$=8.2 Hz); 6.77 (d, 1H, H-2, $J_{1,2}$=8.2 Hz); 3.85 (s, 3H, OCH_3); 3.70 (septet, 1H, C<u>H</u>Me$_2$, J=7.0 Hz); 2.73-2.63 (m, A_2B_2 system, 4H, 2xH-9 ve 2xH-10); 1.41 (d, 6H, CH<u>Me$_2$</u>, J=7.0 Hz).

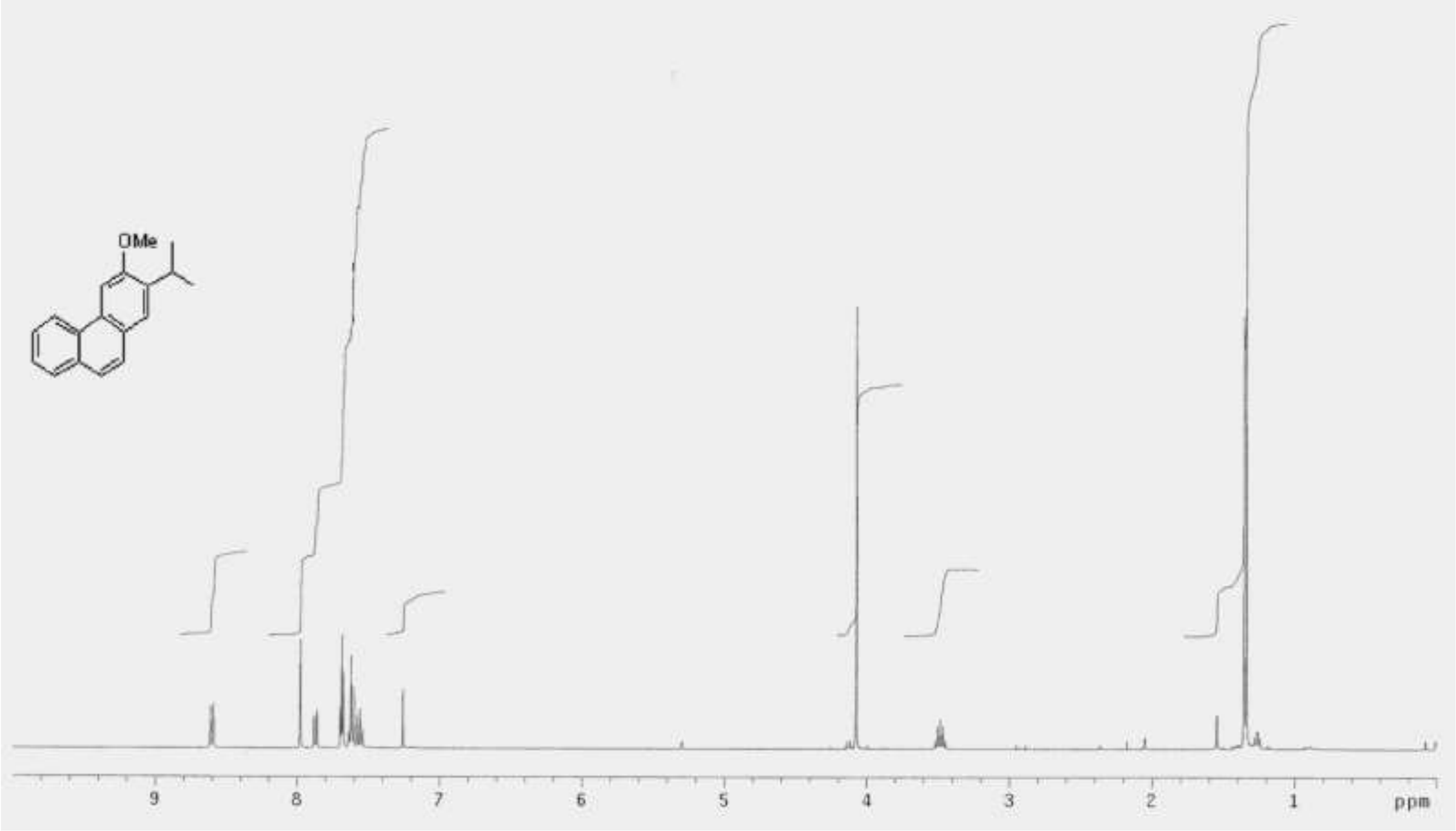

2-isopropyl-3-methoxyphenanthrene (**10**) 400 MHz ^{1}H-NMR Spectrum ($CDCl_3$) δ 8.60 (bd, 1H, H-5, $J_{5,6}$=8.1 Hz); 7.98 (s, 1H, H-1); 7.87 (d, 1H, H-8, $J_{7,8}$=7.7 Hz); 7.69 (s, 1H, H-4); 7.68 (d, 1H, H-9[a], $J_{9,10}$=8.8 Hz); 7.62 (bt, 1H, H-6 and H-10 overlapped); 7.61 (d, 1H, H-10[a], $J_{9,10}$=8.8 Hz); 7.56 (dt, 1H, H-7, $J_{7,8}$=$J_{6,7}$=7.7 Hz, $J_{5,7}$=1.1 Hz); 4.08 (s, 3H, OCH_3); 3.48 (septet, 1H, C<u>H</u>Me_2, J=6.6 Hz); 1.35 (d, 6H, CH<u>Me_2</u>, J=6.6 Hz).

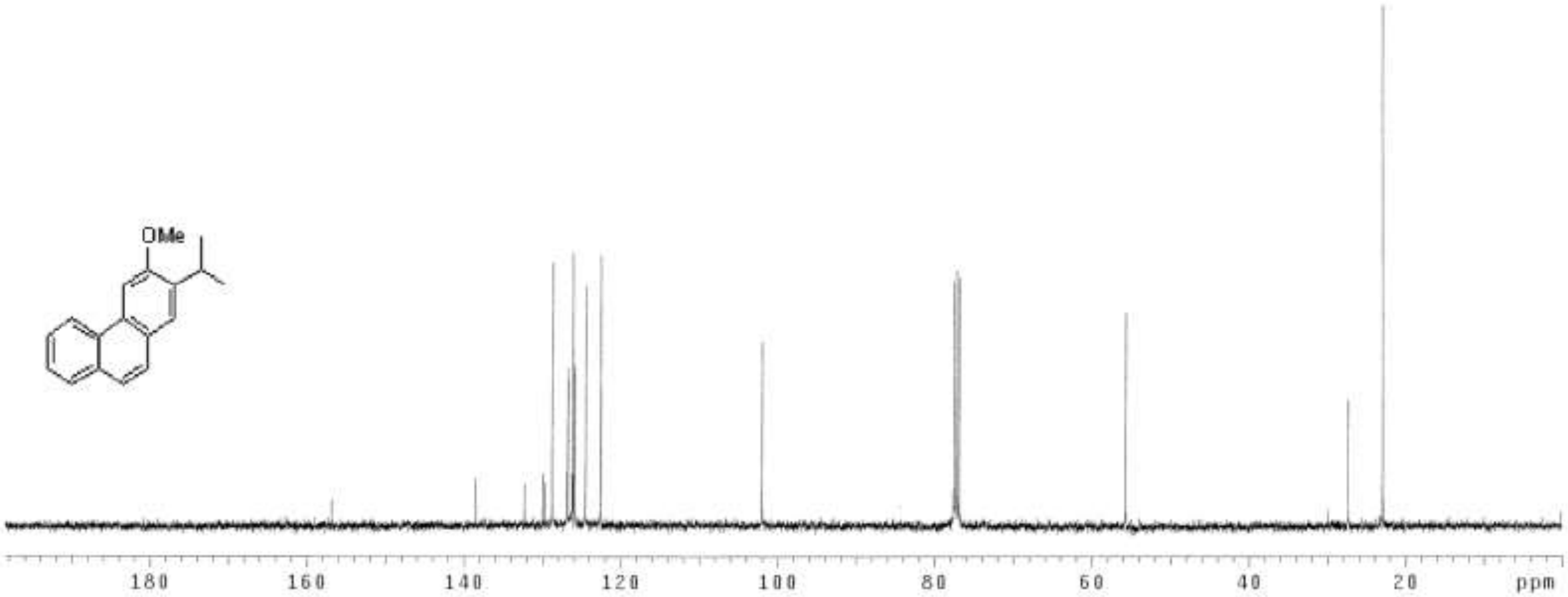

2-isopropyl-3-methoxyphenanthrene (**10**) 100 MHz ^{13}C-NMR Spectrum ($CDCl_3$) δ 156.6 (C-3); 138.3 (s); 132.1 (s); 129.8 (s); 129.5 (s); 128.6 (d); 126.7 (s); 126.6 (d); 126.1 (d); 125.9 (d); 125.7 (d); 124.4 (d); 122.4 (d); 101.8 (d); 55.5 (OCH_3); 27.1 (isopropyl CH); 22.8 ($2xCH_3$).

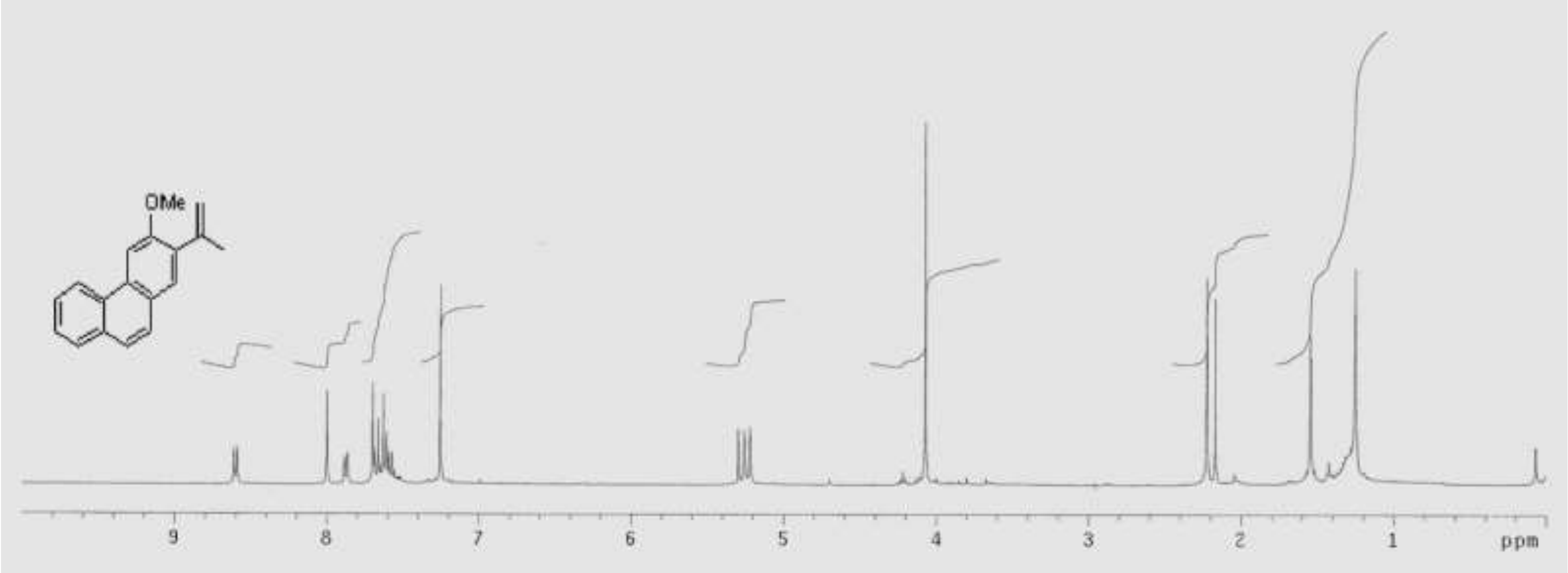

3-methoxy-2-isopropenylphenanthrene (**10a**) 400 MHz ^{1}H-NMR Spectrum ($CDCl_3$) δ 8.60 (d, 1H, H-5, $J_{5,6}$=8.4 Hz); 8.00 (s, 1H, H-1); 7.87 (d, 1H, H-8, $J_{7,8}$=7.7 Hz); 7.70 (s, 1H, H-4); 7.67 (d, 1H, H-9, $J_{9,10}$=9.2 Hz); 7.63 (t, 1H, H-6 and H-10 overlapped); 7.62 (d, 1H, H-10, $J_{9,10}$=9.2 Hz); 7.58 (t, 1H, H-7, $J_{6,7}$=$J_{7,8}$=7.7 Hz); 5.26 (bs, 1H, C=$\underline{CH_2}$); 5.22 (bs, 1H, C=$\underline{CH_2}$); 4.07 (s, 3H, OCH_3); 2.22 (bs, 3H, =C-CH$\underline{_3}$).

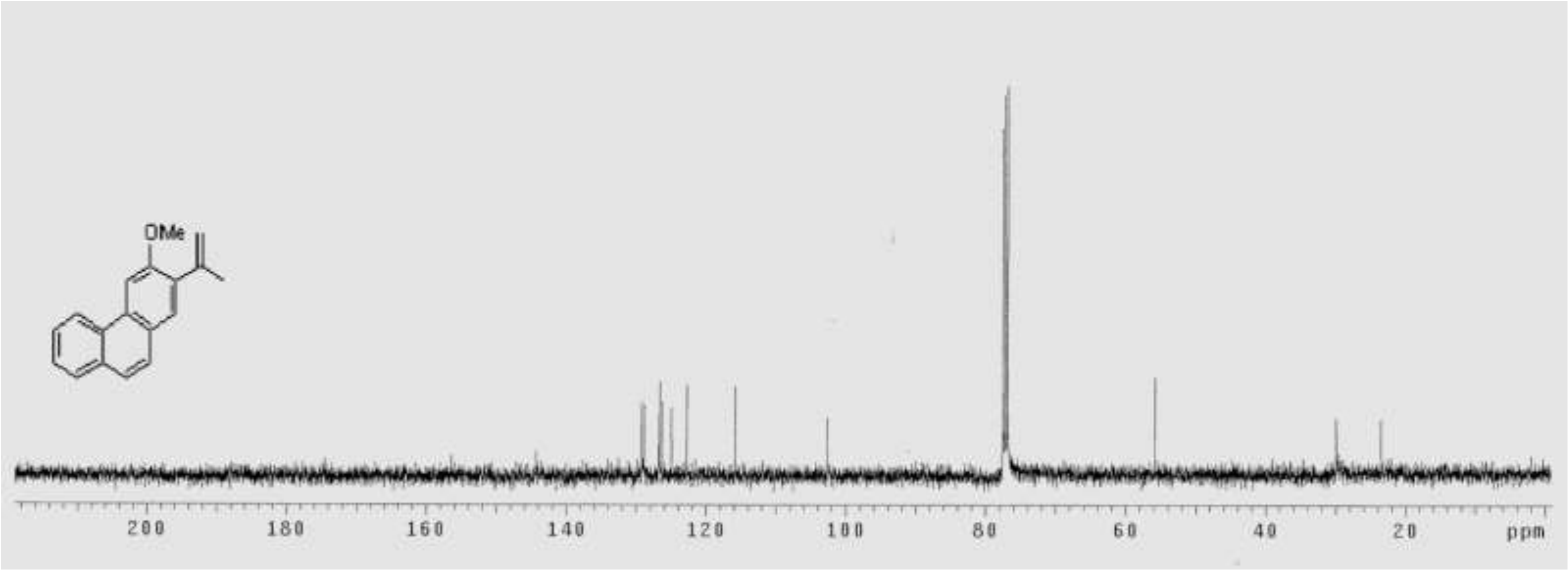

3-methoxy-2-isopropenylphenanthrene (**10a**) 100 MHz ^{13}C-NMR Spectrum ($CDCl_3$)

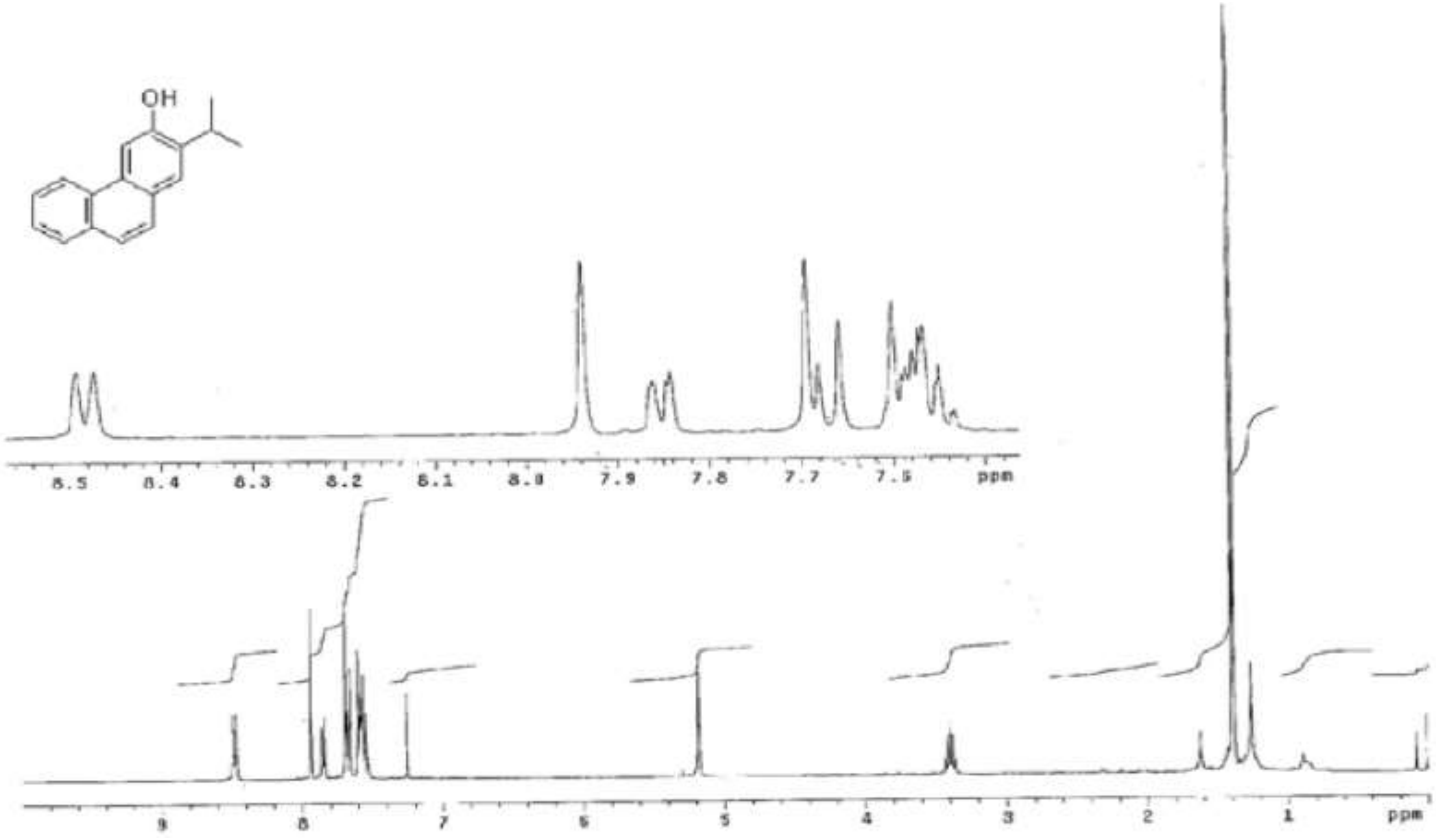

2-Isopropylphenanthren-3-ol **(11)** 400 MHz ^{1}H-NMR Spectrum ($CDCl_3$) δ 8.48 (d, 1H, H-5, J = 7.6 Hz); 7.94 (s, 1H, H-1); 7.85 (bd, 1H, H-8, J = 8.0 Hz); 7.70 (s, 1H, H-4); 7.67 (A part of AB system, d, 1H, H-9, J = 9.3 Hz); 7.59 (B part of AB system, d, 1H, H-10, J = 9.3 Hz); 7.59-7.52 (m, 2H, H-6 and H-7); 5.19 (s, 1H, OH); 3.40 (septet, 1H, $CH(CH_3)2$, J = 7.0 Hz); 1.40 (d, 3H, CH_3, J = 7.0 Hz); 1.39 (d, 3H, CH_3, J = 7.0 Hz).

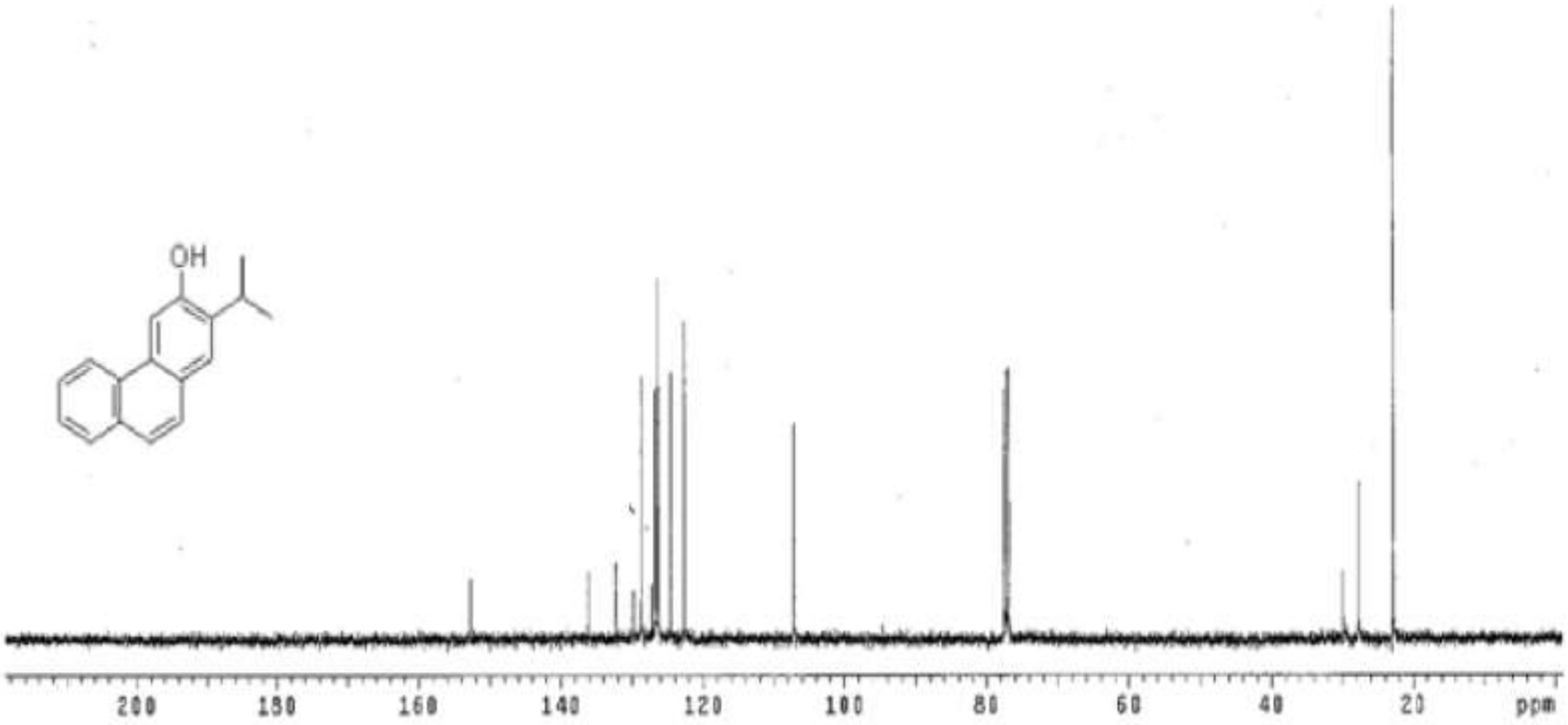

2-Isopropylphenanthren-3-ol **(11)** 100 MHz ^{13}C-NMR Spectrum ($CDCl_3$) δ 152.5 (C-3); 136.0 (s); 132.0 (s); 129.7 (s); 129.4 (s); 128.5 (d); 127.0 (s); 126.6 (d); 126.2 (d); 126.2 (d); 126.0 (d); 124.4 (d); 122.5 (d); 106.9 (d); 27.5 ($CHMe_2$); 22.6 (2x$CHMe_2$).

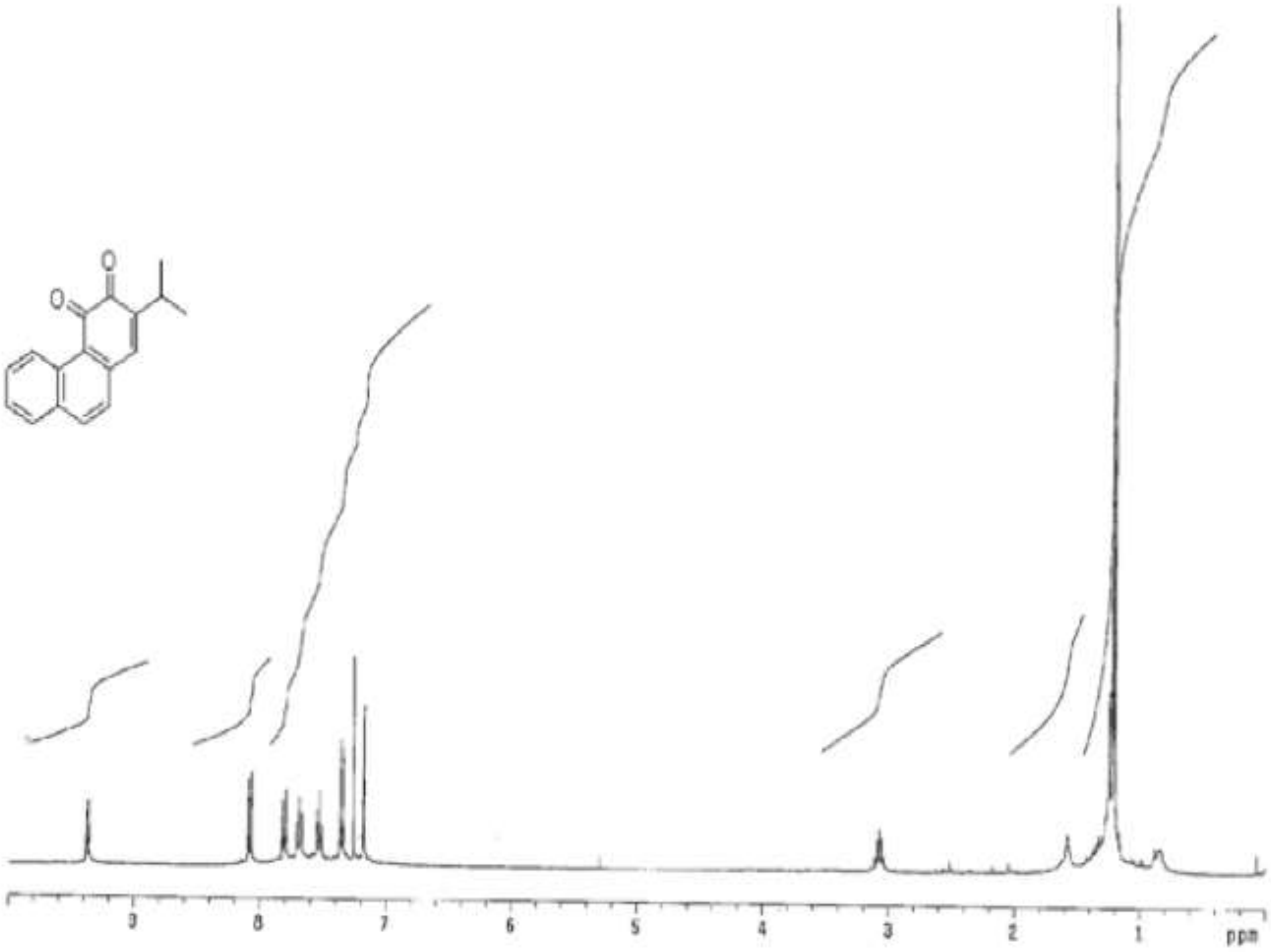

2-Isopropylphenanthren-3,4-dione **(12)** 400 MHz ^{1}H-NMR Spectrum ($CDCl_3$) δ 9.37 (d, 1H, H-5, J = 8.6 Hz); 8.08 (d, 1H, H-8, J = 8.6 Hz); 7.80 (bd, 1H, H-10, J = 8.2 Hz); 7.69 (ddd, quasi bt, 1H, H-6[b], J = 8.6 Hz, J = 6.9 Hz, J = 1.4 Hz); 7.52 (ddd, quasi bt, 1H, H-7[b], J = 8.6 Hz, J = 6.9 Hz, J = 0.9 Hz); 7.35 (d,1H, H-9, J = 8.2 Hz); 7.18 (d, 1H, H-1, J = 0.9 Hz); 3.07 (septet, 1H, $CH(CH_3)_2$, J = 6.9Hz); 1.21 (d, 6H, $CH(CH_3)_2$, J = 6.9 Hz).

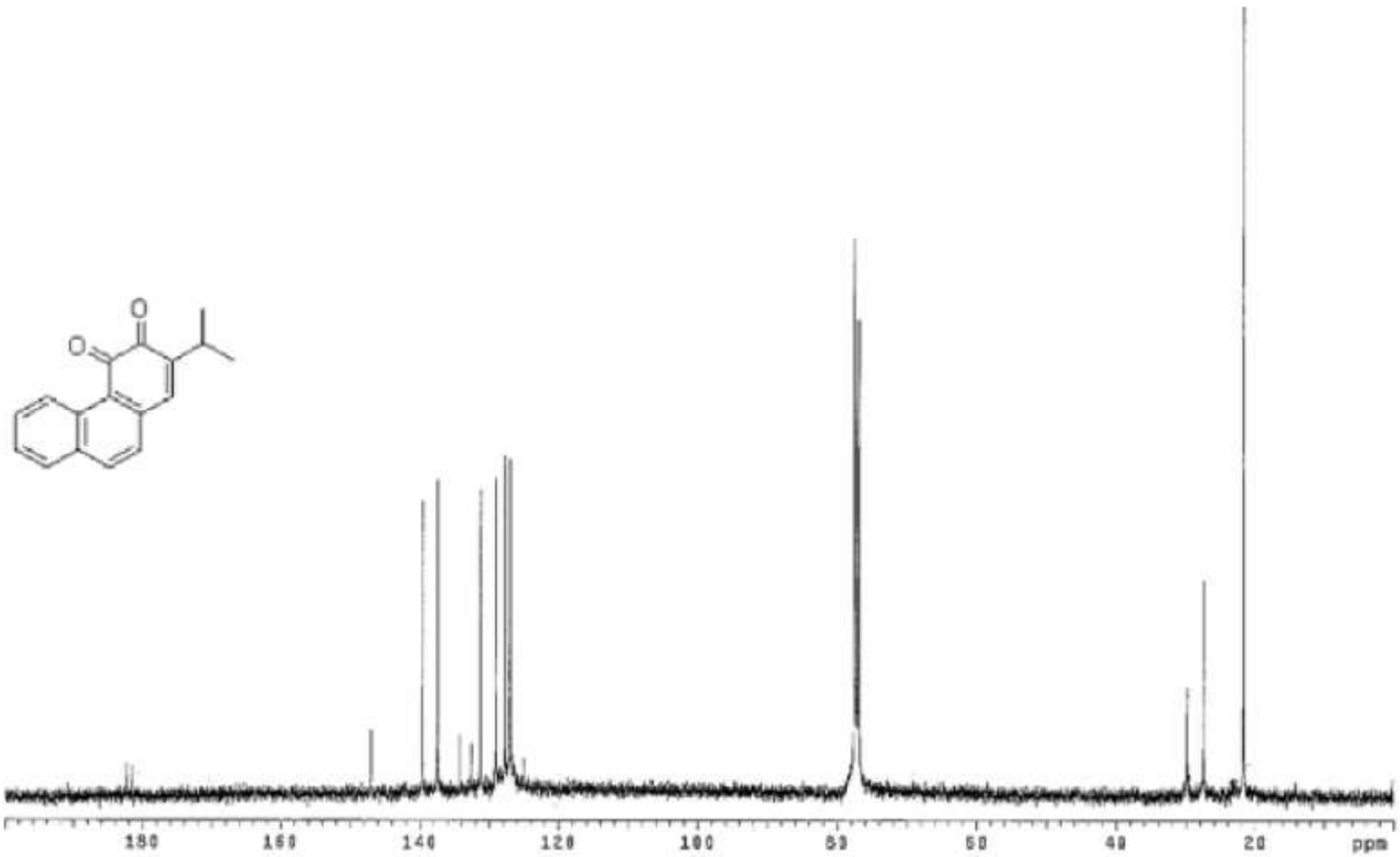

2-Isopropylphenanthren-3,4-dione **(12)** 100 MHz ^{13}C-NMR Spectrum ($CDCl_3$) δ 182.3 (s); 181.5 (s); 146.9 (s); 139.7 (d); 137.5 (d); 134.3 (s); 132.5 (s); 131.3 (d); 129.1 (d); 127.7 (d); 127.1 (d); 126.9 (d); 125.0 (s); 27.4 (d); 21.7 (q).

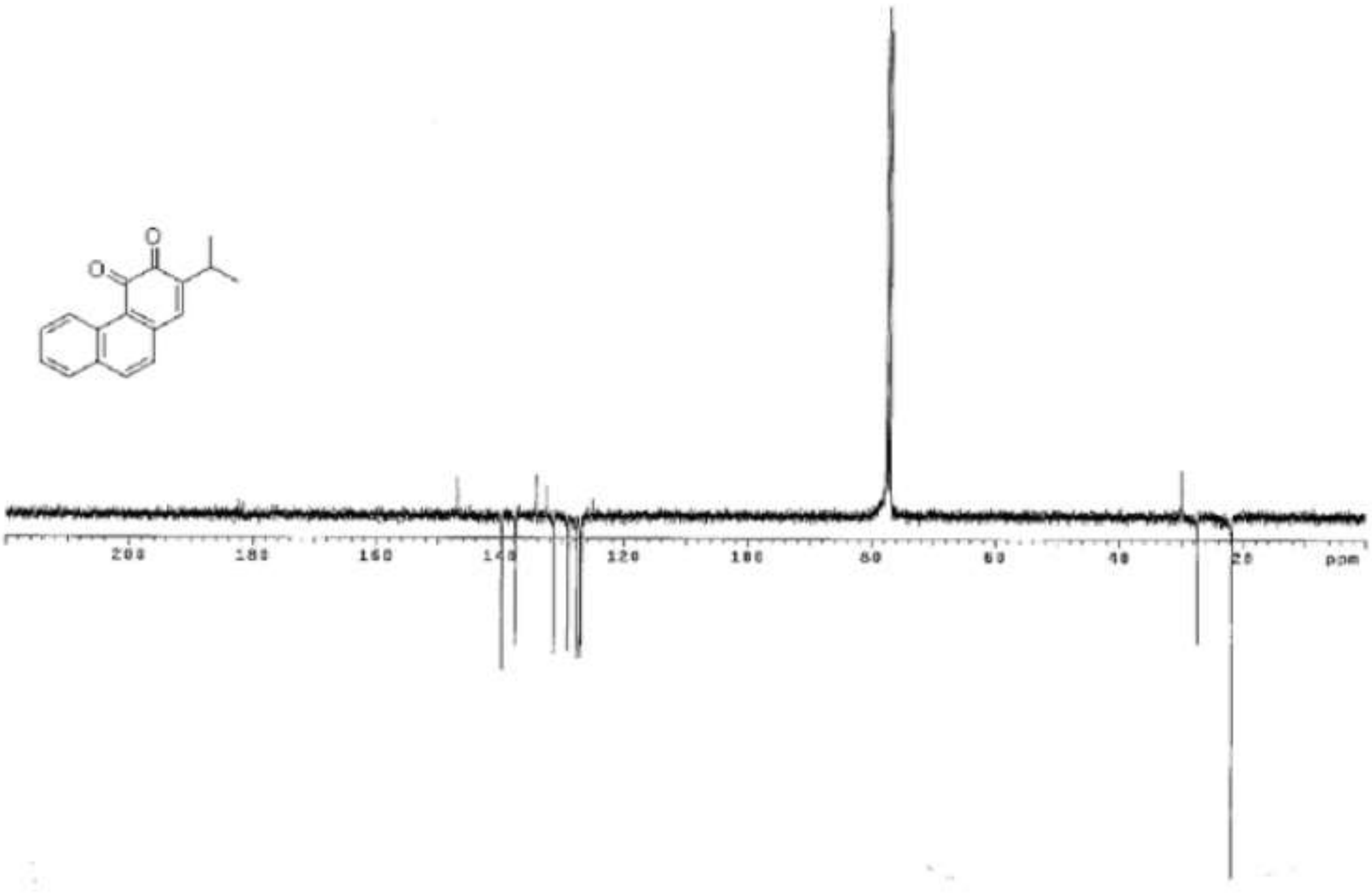

100 MHz APT Spectrum of 2-Isopropylphenanthren-3,4-dione **(12)**
